# An Intelligent Control Strategy for Electromagnetically Driven Control Valve Systems Based on DDPG-PID Auto-Tuning

**DOI:** 10.3390/s26216982

**Published:** 2026-11-04

**Authors:** Wei Li, Shuxun Li, Bojiang Yin, Jian Zhang, Jianlong Zhu, Zhijun Lei

**Affiliations:** 1 School of Petrochemical Engineering, Lanzhou University of Technology, Lanzhou 730050, China; 2 Machinery Industry Pump Special Valve Engineering Research Center, Lanzhou 730050, China

**Keywords:** electromagnetic linear actuator, control valve, reinforcement learning, PID self-tuning, adaptive control

## Abstract

In aerospace, industrial automation, and intelligent equipment, electromagnetically driven valve control systems often suffer from low control accuracy and stalling during switching and servo operations because of strong nonlinearity, parameter uncertainty, frequent operating condition variations, and the empirical nature of conventional PID tuning. To address these issues, this study proposes a PID self-tuning control method based on Deep Deterministic Policy Gradient (DDPG) to improve the dynamic response and robustness of valve control systems. An electromagnetically driven control valve system suitable for both on/off valve modes is constructed, and an interaction framework between the reinforcement learning agent and the Simulink simulation environment is established. The proposed reinforcement-learning control strategy establishes a mapping from the measured system states to the PID gains, enabling the gains to be adaptively adjusted according to the current operating state. To improve training stability and sample efficiency, experience replay and target network soft updates are introduced, and an Actor–Critic architecture is adopted for policy learning and value evaluation. The proposed strategy is further implemented in both the simulation model and MCU controller software, and its performance is compared with traditional PID and fuzzy PID methods. Results show that, in on/off valve mode, the method achieves timed opening, holding, and closing within a preset cycle, while in servo valve mode, it enables accurate position control over a 0~20 mm valve core stroke, with a positioning error of 0.04 mm and a response time below 50 ms. Compared with traditional methods, it shows clear advantages in accuracy, response speed, and adaptability.

## 1. Introduction

With the continuous development of high-end equipment, process industry and intelligent fluid control technology, valve control systems are rapidly evolving towards high response, high precision, high reliability and intelligence [1]. As a key execution unit for realizing flow, pressure and displacement regulation, electromagnetic drive valve control systems are widely used in industrial automation, aerospace, vehicle engineering and intelligent equipment due to their advantages such as compact structure, fast response speed and easy electronic control integration. However, in actual operation, such systems are usually affected by factors such as magnetic circuit saturation, friction and dead zone, time-varying parameters, load disturbance and strong nonlinearity, which leads to complex dynamic characteristics and difficulty in accurate modeling, and thus puts forward higher requirements for the speed, steady-state accuracy and anti-disturbance performance of control algorithms [2,3,4]. In recent years, research on valves and electro-hydraulic servo execution systems has shown that high-performance control under complex working conditions has become an important development direction in this field, especially under the conditions of gap nonlinearity, unknown disturbance and sensor measurement range; thus, the optimization and upgrading of system control strategy has outstanding engineering value [5,6,7].

Currently, PID control remains the most commonly used basic algorithm in electromagnetic drive valve control systems due to its simple structure, convenient engineering implementation, and rich experience in parameter adjustment [8]. However, since electromagnetic actuators for valves typically exhibit obvious nonlinear and time-varying characteristics, fixed-parameter PID often struggles to balance speed and robustness under a wide range of operating conditions [9]. Ramali et al. [10] used a PID controller to regulate the FES system. Bozkurt et al. [11] used a model-based PID control method to regulate the speed of the PMSM and to precisely regulate the permanent magnet synchronous motor. Atam et al. [12] proposed a new gain-scheduled PID control design method, using advanced controllers such as linear parameter variation controllers to optimize PID parameters. Camacho et al. [13] proposed a weighted fractional-order PID controller, which enhances the traditional fractional-order PID structure by introducing explicit tuning term weighting factors. Although the researchers mentioned above have made certain achievements in different fields, PID control is essentially a linear control method based on error feedback. When the system has strong nonlinearity, time-varying characteristics, parameter disturbances or strong external disturbances [14], it is often difficult to maintain the ideal control effect by simply using PID, and problems such as decreased control performance are likely to occur.

More and more researchers are beginning to try to gradually expand from traditional PID control to higher-level methodologies such as intelligent control, robust control and data-driven control [15]. Among them, fuzzy control, fuzzy PID composite control and adaptive control are widely used in electromagnetic actuators because they have a weak dependence on the precise model of the object and a strong adaptability to parameter perturbations and external disturbances; sliding mode control, robust control and feedforward-feedback composite control have shown obvious advantages in high-response and high-precision valve drive scenarios due to their strong anti-uncertainty ability and dynamic tracking ability [16]. With the continuing development of embedded computing platforms, model-based approaches such as model predictive control and extended-state-observer-based output-feedback control have been applied to control-valve and hydraulic-actuator systems to address valve nonlinearities, input constraints, unmeasured states, and external disturbances [17,18,19].

However, compared with the above methods that rely on prior models, empirical rules or local parameter tuning, reinforcement learning, as a data-driven intelligent control method for sequential decision problems, has shown more prominent development potential in the field of control systems in recent years [20]. This method constructs a “state-action-reward” interaction mechanism, enabling the controller to learn the optimal control strategy autonomously in continuous interaction with the environment without relying entirely on precise mathematical models. Therefore, it is particularly suitable for valve electromechanical conversion devices with magnetic circuit nonlinearity, friction dead zone, time-varying parameters and frequent switching of operating conditions [21]. Furthermore, reinforcement learning can be combined with deep neural networks to approximate high-dimensional state spaces and complex nonlinear mapping relationships, enhancing the system’s adaptive adjustment capability to load fluctuations, medium pressure changes and temperature rise effects [22,23]. Xiang et al. [24] proposed a hierarchical reinforcement learning strategy for the collaborative control problem of cross-domain heterogeneous unmanned systems in water and air, which improved the fault tolerance capability of the system. Su et al. [25] conducted simulation analysis and experimental verification of the SAC-ARC control strategy based on the heterogeneous dual-valve coordinated control strategy SAC-ARC, which integrates reinforcement learning and the adaptive robust control algorithm. Yu et al. [26] proposed a hybrid control algorithm based on reinforcement learning and proportional derivative, which realized the effective control of the aircraft. Zhang et al. [27] proposed a novel multi-agent reinforcement learning behavior control (MARLBC) method to reduce the dependence on human intelligence and high-performance computing hardware. Niu et al. [28] studied the obstacle avoidance task of the intelligent agent based on the MADDPG deep reinforcement learning algorithm. Wang et al. [29] described the latest progress and future research direction of automatic ship control based on networking and deep reinforcement learning. Yu et al. [26] proposed a method combining reinforcement learning with proportional-derivative control; simulation results demonstrate that this method significantly improves the flight stability of micro aerial vehicles. Li et al. [30] proposed two control algorithms based on reinforcement learning, which effectively solved the data-driven optimal control problem in a continuous-time linear non-zero sum game.

In summary, this study proposes a reinforcement learning-based PID parameter self-tuning control strategy (DDPG-PID Auto-Tuning) based on deep deterministic policy gradient. The proposed strategy does not require an exact analytical model to be explicitly incorporated into the online PID control law. However, an approximate model that captures the dominant dynamics of the valve system is required to construct the Simulink environment for offline policy training. By introducing an experience replay mechanism and a soft update method for the target network, sample utilization efficiency is improved, policy oscillations during training are suppressed, and the stability of the learning process is enhanced. Meanwhile, the Actor–Critic architecture provides a clear value assessment basis for policy iteration, making it more efficient than traditional direct search-based parameter tuning methods such as grid search and randomized trials. Based on this, the reinforcement learning strategy is combined with PID control to achieve adaptive tuning of PID parameters, enabling precise adjustment of different valve spool stroke setpoints and providing an important reference for precise valve spool stroke adjustment in electromagnetic control valves.

The main contributions of this paper are as follows:

1. This paper proposes a PID gain tuning strategy based on DDPG. Although offline policy training requires an approximate Simulink model, the trained Actor can adaptively generate bounded PID gains based on measured system states, thereby reducing reliance on manual PID tuning and online model calculations.
2. An online mapping mechanism between system state and PID parameters was established, enabling the controller to adjust control parameters in real time according to the operating state, thereby improving the dynamic performance and robustness of the system under nonlinear, parameter uncertainty, and changing operating conditions.
3. The proposed controller was integrated into the simulation model and MCU system software, completing the entire research process from algorithm design and simulation verification to embedded implementation, thus verifying the engineering feasibility of the proposed method in valve control systems.

## 2. Preliminaries

### 2.1. PID Control Principle

**Figure 1:**
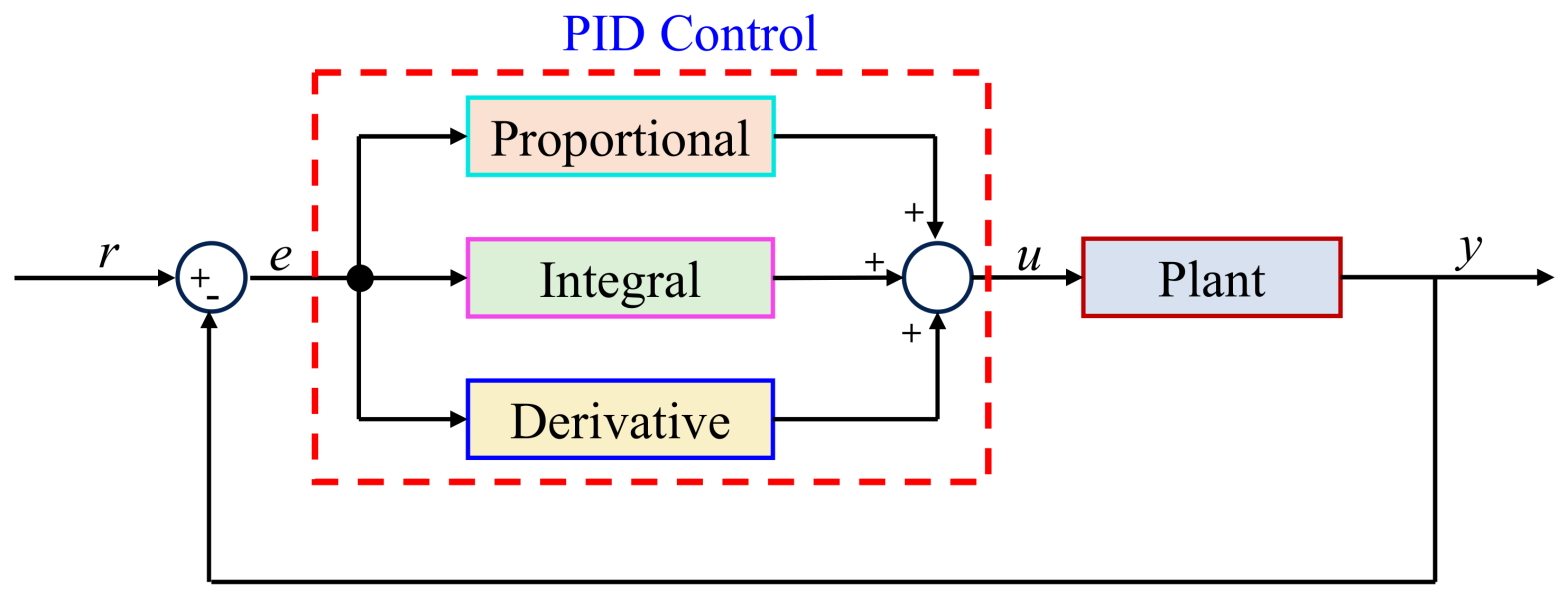
PID control system structure.

The PID (proportional–integral–derivative) controller is a classical feedback control method widely used in automation systems because of its simple structure, robustness, and ease of implementation [31,32]. In this study, PID control is adopted as the basic control framework, and its parameters are further optimized by the proposed DDPG-PID Auto-Tuning strategy. The controller output is determined by the weighted combination of the proportional, integral, and derivative terms, where the proportional gain ($K_{p}$) mainly affects the response speed, the integral gain ($K_{i}$) helps eliminate steady-state error, and the derivative gain ($K_{d}$) improves dynamic response and suppresses overshoot. Figure 1 shows the structure of the PID controller in a closed-loop control system.

### 2.2. Reinforcement Learning Theory

**Figure 2:**
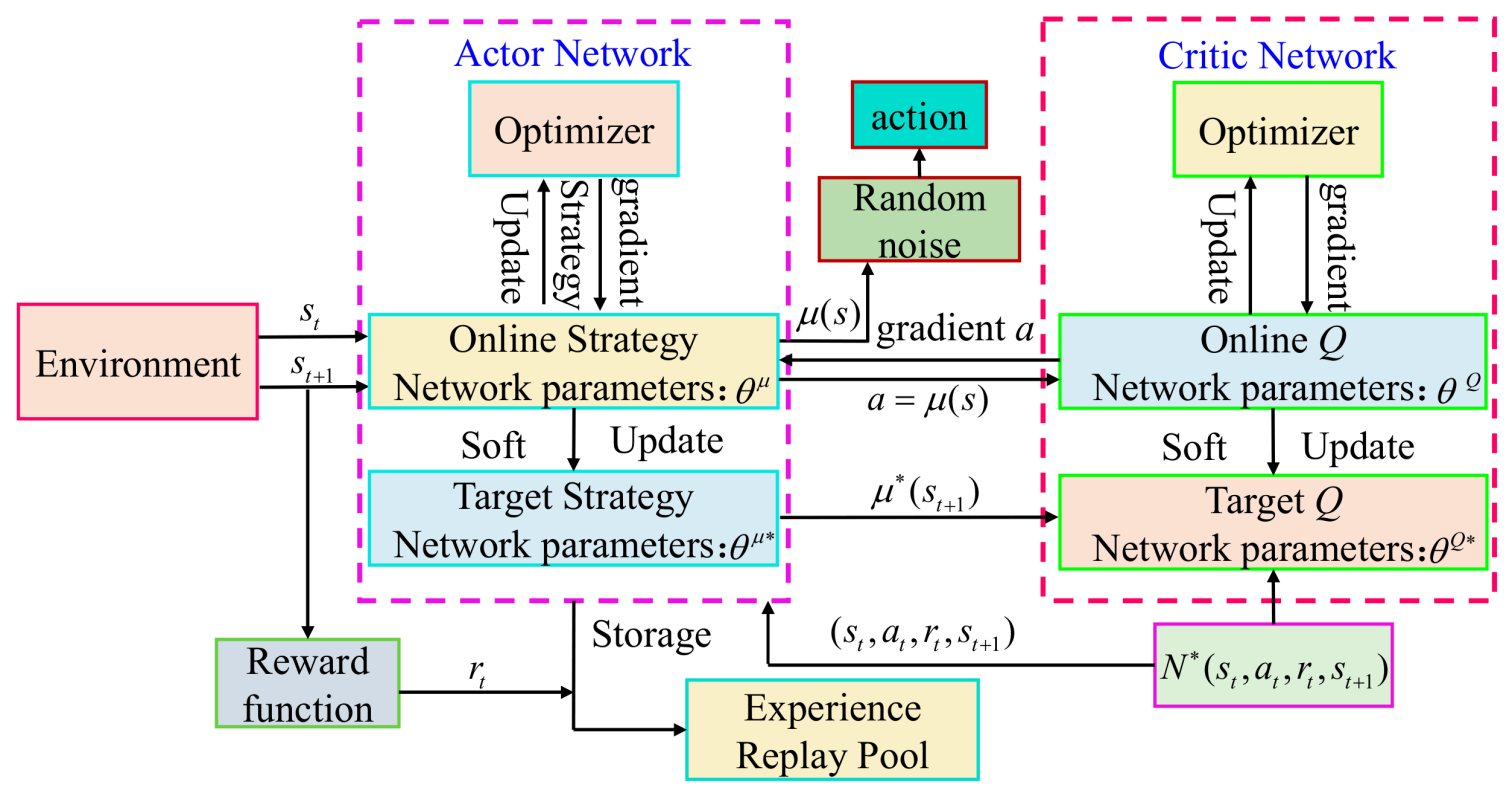
DDPG network structure.

Reinforcement learning (RL) is a machine learning method that learns decision-making strategies through interaction with the environment. The agent optimizes its strategy based on the reward signal fed back by the environment through exploration and trial and error [33,34]. Compared with traditional reinforcement learning algorithms (such as Q-learning), the DDPG (Deep Deterministic Policy Gradient) algorithm has shown significant advantages in dealing with problems in continuous action space. DDPG is based on the actor–critic architecture and uses deep neural networks to approximate the policy and value function, which can efficiently learn complex control tasks [35]. Its core advantage is that it directly outputs continuous action values, avoiding the discretization problem faced by Q-learning in continuous action space. Especially in the solenoid valve stroke control, the nonlinear relationship between the solenoid valve position and the control signal makes it difficult for traditional PID controllers to cope with changing working conditions, while DDPG can automatically optimize PID parameters through repeated interaction with the environment, overcoming the limitations of manual adjustment [36]. The target network and experience playback mechanism of DDPG ensure the stability of training, enabling it to provide accurate and robust control strategies when facing complex and uncertain control environments. Therefore, the application of the DDPG algorithm in solenoid valve stroke control can not only achieve adaptive adjustment of PID parameters, but also maintain efficient control performance under complex working conditions, which has significant advantages over traditional control methods [37]. Figure 2 shows a schematic diagram of the structure of the DDPG algorithm.

Figure 2 illustrates the basic framework of the DDPG algorithm, which combines deep learning and reinforcement learning to effectively solve decision-making problems in continuous action spaces. DDPG belongs to an “Actor-Critic” structure, which includes an Actor network and a Critic network, which perform policy updates and value evaluation tasks, respectively.

State $s_{t}$ and reward $r_{t}$ are generated interactively from the current environment state:

(1) $$\left(s_{t+1},r_{t})=\mathrm{Environment}(s_{t},a_{t}\right)$$

where $s_{t}$ represents the current environment state, and $a_{t}$ is given by the Actor network.

The goal of the Actor network is to generate action $a_{t}$ through policy function $\mu (s_{t};\theta^{\mu })$.

(2) $$a_{t}=\mu (s_{t};\theta^{\mu })+\mathrm{random}\;\mathrm{noise}$$

The Critic network calculates the current *Q*-value using states $s_{t}$ and $a_{t}$:

(3) $$Q(s_{t},a_{t};\theta^{Q})=\mathrm{Critic}\;\mathrm{Network}(s_{t},a_{t};\theta^{Q})$$

where $\theta^{Q}$ represents the Critic network parameters.

## 3. The Proposed Method

Section 2 introduced the theory of PID control and the relevant knowledge of reinforcement learning. This section will further explore the implementation process of the constructed reinforcement learning-based PID control strategy.

### 3.1. The Proposed DDPG-PID Auto-Tuning Control Strategy

This section details the self-tuning PID control strategy based on the Deep Deterministic Policy Gradient (DDPG) algorithm proposed in this study, and how it is applied to a control valve system directly driven by an electromagnetic linear actuator. This control strategy automatically optimizes the PID parameters ($K_{p}$, $K_{i}$, $K_{d}$) through reinforcement learning, achieving more precise and efficient control. The model interacts with the control valve system in the Simulink environment, dynamically adjusting the PID parameters based on real-time feedback of the control error and its changes, ensuring high control performance under different operating conditions. In this model, the state space includes key state variables such as the control error and its changes, while the action space consists of the three gains of the PID controller, which are limited within a set range to ensure system stability and safety.

**Figure 3:**
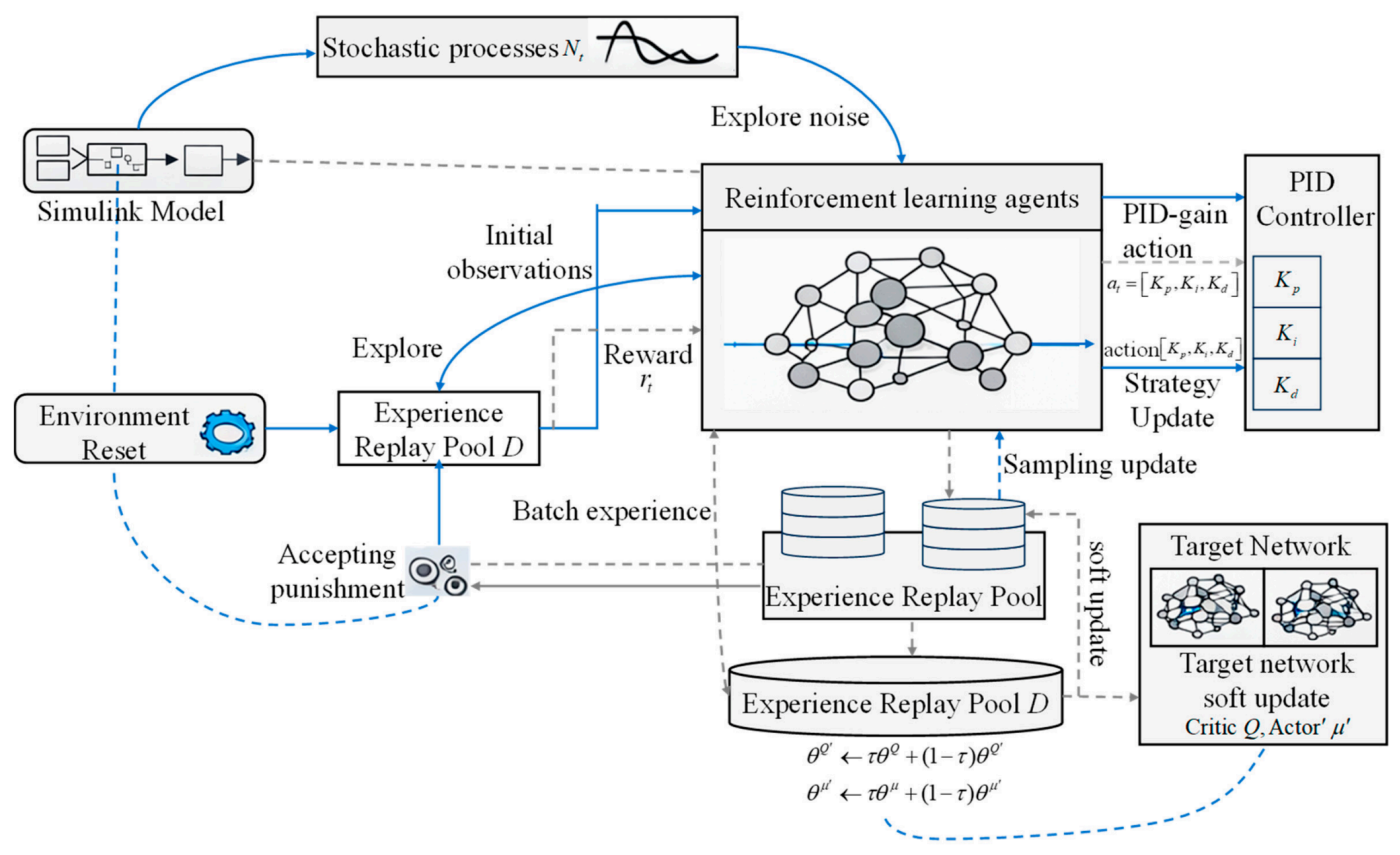
DDPG-PID Auto-Tuning Control Strategy Structure.

The agent optimizes the control strategy using reinforcement learning techniques such as experience replay and soft updates of the target network. During training, the Critic network evaluates the value of state–action pairs, while the Actor network generates corresponding PID parameters based on the current state. This method effectively improves the diversity of training and the generalization ability of the agent by introducing randomization of the reference signal and initial state, avoiding overfitting. Figure 3 illustrates the structure of the DDPG-PID Auto-Tuning control strategy established in this study.

As shown in the diagram, the model initializes observations and explorations through a stochastic process, generating noise. This noise is input into the reinforcement learning agent, which continuously updates its policy to adjust the parameters $K_{p}$, $K_{i}$, and $K_{d}$ of the PID controller to optimize the system. An experience replay pool *D* stores the agent’s experience; the agent updates its policy based on batch experience and adjusts the PID controller’s actions according to environmental feedback. The target network continuously performs soft updates to help the agent make better predictions and decisions. During the control process, the policy is continuously optimized. Through continuous experience sampling and updates to the replay pool, the parameters of the PID controller are adjusted more precisely, thus achieving system self-optimization.

To ensure the reproducibility of the reinforcement learning control problem, this study further specifies the composition of the reward function. At time step *t*, the reward function is defined as

(4) $$R_{k}=-\omega_{e}{\tilde{e}}_{k}^{2}-\omega_{\Delta e}{\left(\Delta {\tilde{e}}_{k}\right)}^{2}-\omega_{u}{\tilde{u}}_{k}^{2}-\omega_{K}{\left\|\Delta {\tilde{K}}_{k}\right\|}_{2}^{2}-\omega_{o}{\tilde{o}}_{k}^{2}+r_{b}\mathrm{II}(\left|e_{k}\right|\leq \varepsilon )$$

Among them, when the spool stroke is 20 mm, the displacement tracking error and the rate of change in the error are as follows:

(5) $$e_{k}=x_{k}^{\mathrm{ref}}-x_{k},\;{\tilde{e}}_{k}=\mathrm{clip}(\frac{e_{k}}{20},-1,1)$$

(6) $$\Delta e_{k}=e_{k}-e_{k-1},\;\Delta {\tilde{e}}_{k}=\mathrm{clip}(\frac{\Delta e_{k}}{20},-1,1)$$

If $u_{k}$ is a PWM command with a range of 0–1000:

(7) $${\tilde{u}}_{k}=\mathrm{clip}(\frac{u_{k}}{1000},0,1)$$

The PID response is normalized as follows:

(8) $${\tilde{K}}_{p,k}=2\frac{K_{p,k}-80}{103-80}-1$$

(9) $${\tilde{K}}_{i,k}=2\frac{K_{i,k}-18.3}{20-18.3}-1$$

(10) $${\tilde{K}}_{d,k}=2\frac{K_{d,k}-3.5}{5-3.5}-1$$

The change in gain is

(11) $$\Delta {\tilde{K}}_{k}={\tilde{K}}_{k}-{\tilde{K}}_{k-1}$$

For a fixed-stroke response where the target stroke increases from a smaller value to a larger one, the normalized overshoot can be defined as

(12) $${\tilde{o}}_{k}=\frac{\max(0,x_{k}-x_{k}^{\mathrm{ref}})}{e_{\max}},\;e_{\max}=20\mathrm{mm}$$

The indicator function is defined as

(13) $$\mathrm{II}(\left|e_{k}\right|\leq \varepsilon )=\left\{\begin{array}{l} 1,\;\left|e_{k}\right|\leq \varepsilon \\ 0,\;\left|e_{k}\right|>\varepsilon \end{array}\right.$$

where $R_{k}$ is the instantaneous reward at time step *k*, $x_{k}^{\mathrm{ref}}$ is the target spool displacement, $K_{k}$ is the PID gain vector, ε is the allowable spool displacement error, and ${\left|\cdot \right|}_{2}$ is the Euclidean norm of the vector.

Table 1 lists the specific values for the reward function weights in the aforementioned formula.

Based on the above, Algorithm 1 summarizes the key steps of the DDPG-PID Auto-Tuning control strategy.
**Algorithm 1** DDPG-PID Auto-Tuning Control Strategy**Initialize** Simulink model DDPG-PID Auto-Tuning. Define a reinforcement learning environment, including a state space and an action space, where the action space consists of the gain parameters $K_{p}$, $K_{i}$, and $K_{d}$ of the PID controller. **Initialize** two Critic networks $\phi_{1}$ and $\phi_{2}$, Actor network $\mu (s\left|\theta^{\mu }\right.)$, and their corresponding target networks $\mu^{\prime }$, $\phi_{1}^{\prime }$, and $\phi_{2}^{\prime }$. **Initialize** the prioritized experience replay buffer *D*. **for** episode = 1, *M* performs the following steps  Initialize the random process for action exploration *N*  Receive initial observation state $s_{1}$  **for** t = 1, *T* performs the following steps  Select the action according to the current policy:
(14)$$a_{t}=\mu (s_{t}\left|\theta^{\mu }\right.)+N_{t}$$
  where $a_{t}$ represents PID parameters $K_{p}$, $K_{i}$, and $K_{d}$.   Execute action $a_{t}$, which means using the current PID gain $K_{p}$, $K_{i}$, and $K_{d}$ control model.  Observe the reward $r_{t}$, next state $s_{t+1}$, and the termination signal is_done.  Store experience $\left(s_{t},a_{t},r_{t},s_{t+1},\mathrm{is}\text{\_}\mathrm{done}\right)$ in the replay buffer *D*.  Calculate the TD error $\delta_{t}$ and priority $p_{t}$, and sample *N* experiences according to the prioritized sampling probability p(i) using Equations (21)–(24).  Calculate the two target values $y_{t}^{\mathrm{critic}1}$ and $y_{t}^{\mathrm{critic}2}$ using Equations (28) and (29).  Update Critic networks $\phi_{1}$ and $\phi_{2}$ according to Equations (30) and (31).  Update the Actor network according to the deterministic policy gradient in Equation (27).  Update the priorities of the sampled experiences using the new TD errors according to Equations (19) and (20).  Soft-update the target Actor $\mu^{\prime }$ and the target Critic networks $\phi_{1}^{\prime }$ and $\phi_{2}^{\prime }$.  Set $s_{t}\leftarrow s_{t+1}$.  **if** is_done = 1, **break**.  End the current round and continue to the next round.  After training, simulation verification is performed.  The trained agent was used for simulation to verify the effectiveness of the PID parameter self-tuning controller. During the verification process, the agent optimized the control system response by adjusting the PID gains $K_{p}$, $K_{i}$, and $K_{d}$. **end** **end**

### 3.2. Controller Network Design

**Figure 4:**
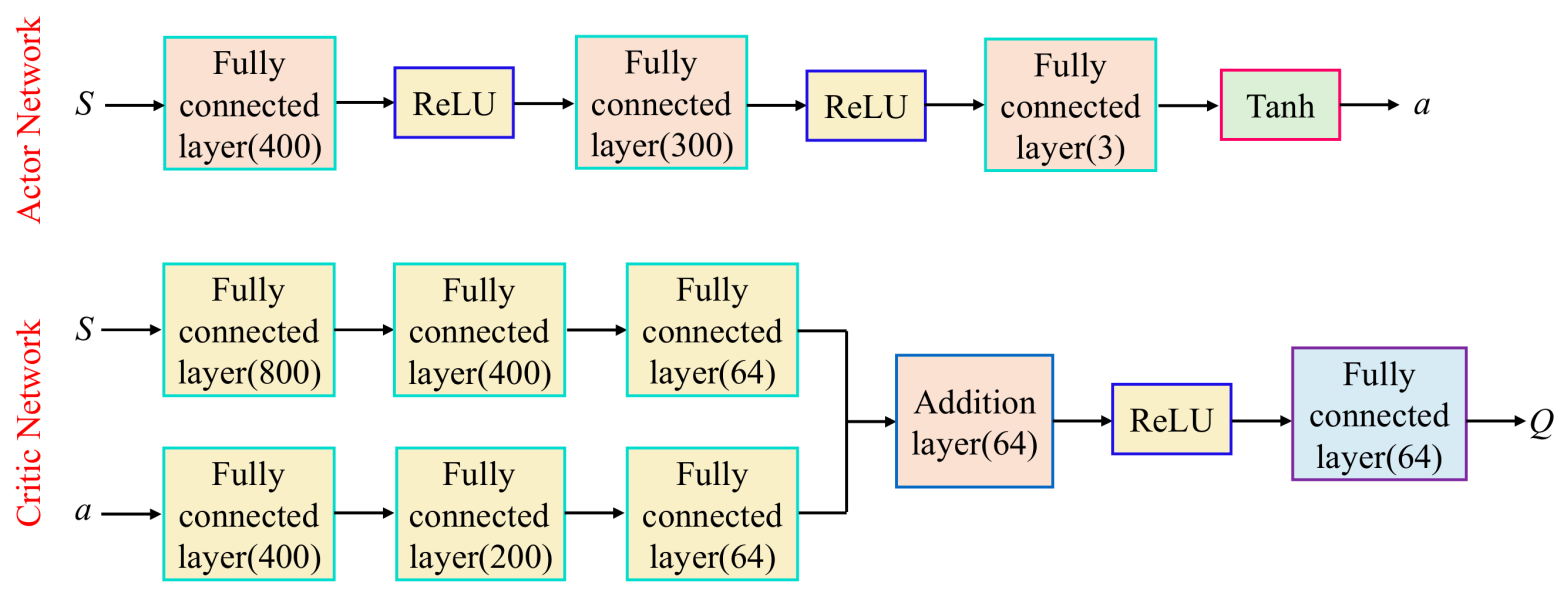
DDPG network structure diagram.

Methods based on DDPG-PID have been investigated for control targets such as aircraft and pumps; however, their application to the position control of electromagnetic control valves remains relatively limited. Therefore, this study develops a DDPG-PID self-tuning control strategy for the electromagnetic control valve under test. Figure 4 shows the DDPG network structure. In the figure, the Actor network is responsible for outputting action *a* based on the input state *S*, i.e., the parameters $K_{p}$, $K_{i}$, and $K_{d}$ of the PID controller. It consists of three fully connected layers, including 400, 300, and 3 neurons, respectively, and undergoes a nonlinear transformation using the ReLU activation function. Finally, the output is mapped to the range [−1, 1] using the Tanh activation function. The Critic network is responsible for evaluating the value function *Q* of the current policy. Its input includes the state *S* and action *a*. The state input passes through three fully connected layers with 800, 400, and 64 neurons, respectively, and the action input passes through three fully connected layers with 400, 200, and 64 neurons, respectively. The two are then combined through an additive layer, passed through the ReLU activation function, and finally output through a fully connected layer to produce the value function *Q*. The Actor network optimizes the control policy through feedback from the Critic network, ultimately achieving automatic adjustment of the PID parameters. The online neural network computation consists of only one forward inference operation of the Actor network. For a network structure of 3-400-300-3, a single inference operation requires approximately 122,100 multiply–accumulate operations, encompassing 122,803 trainable parameters. Compared to Actor inference, the subsequent discrete PID calculations require significantly fewer arithmetic operations.

In the Actor network part, the system state *S* is taken as input, and the corresponding valve core stroke $a_{s}$ is output. The system state $S^{\prime }$ is updated to obtain the reward *r*. The specific calculation is as follows:

(15) $$a_{s}={\omega_{ea2}}^{T}\sigma ({\omega_{ea1}}^{T}s-b_{ea})+N$$

where $a_{s}\in R$ is the valve core stroke [0–20 mm], $\omega_{ea2}\in R^{m\times l}$ and $\omega_{ea1}\in R^{m\times n}$ are weight matrices, *N* is Gaussian noise, and $b_{ea}$ is the bias column vector.

Among them, the state vector $s_{t}$ is

(16) $$s_{t}={\left[{\tilde{e}}_{t},\Delta {\tilde{e}}_{t},{\tilde{x}}_{t}\right]}^{T}$$

The Actor network takes *S* and $a_{s}$ as inputs and an evaluation function $Q(s,a_{s})$ as output. The specific calculation is shown below:

(17) $$Q(S,a_{s})={\omega_{ec2}}^{T}\sigma ({\omega_{ec1}}^{T}\left[\begin{array}{l} V_{s}\\ V_{a_{s}} \end{array}\right])$$

(18) $$V_{s}={\omega_{ec5}}^{T}\sigma ({\omega_{ec2}}^{T}\sigma ({\omega_{ec3}}^{T}S-b_{ec2})-b_{ec3})$$

(19) $$V_{a_{s}}={\omega_{ec8}}^{T}\sigma ({\omega_{ec7}}^{T}\sigma ({\omega_{ec6}}^{T}S-b_{ec4})-b_{ec5})$$

where $\omega_{ec1}$, $\omega_{ec3}$, $\omega_{ec4}$, $\omega_{ec5}$, $\omega_{ec6}$, $\omega_{ec7}$ and $\omega_{ec8}$ are all weight matrices; $\omega_{ec2}$ is a weight column vector; and $b_{ec1}∼b_{ec5}$ is a bias column vector.

The state vector at time step *t* consists of the normalized displacement error, the error change, and the actual valve core displacement, and the action vector is

(20) $$a_{t}={\left[K_{p}(t),K_{i}(t),K_{d}(t)\right]}^{T}$$

In the policy update section of Figure 4, the Critic network uses backpropagation to calculate the error:

(21) $$J_{ec}=\frac{1}{q}\sum_{i=1}^{q}{\left(y_{i}-Q(S_{i},a_{si})\right)}^{2}$$

where *q* is the number of samples, and $y_{i}$ is the target evaluation function.

Among them

(22) $$y_{i}=\left\{\begin{array}{l} r_{i}\;\mathrm{is}\_\mathrm{done}=1\\ r_{i}+\gamma Q(S^{\prime },a_{s}^{\prime })\;\mathrm{is}\_\mathrm{done}=0 \end{array}\right.$$

where γ is the discount factor, and $r_{i}$ is the reward.

Meanwhile, the Actor network uses backpropagation to calculate errors:

(23) $$J_{ea}=-\frac{1}{q}\sum_{i=1}^{q}Q_{c}(S_{i},a_{si})$$

### 3.3. Algorithm Improvement

Standard DDPG uses uniform random sampling from the replay buffer and estimates the action value using a single Critic network. Consequently, transitions that are more informative for policy learning may be insufficiently sampled, while approximation errors in the Critic may result in overestimated Q-values and unstable training. To address these issues in the present PID gain-tuning task, prioritized experience replay and a dual-Critic Q-value estimation mechanism are incorporated into the DDPG framework.

In traditional experience replay, all experiences are randomly selected and stored in an experience pool, from which the agent randomly samples during training. However, this method may overlook experiences crucial for policy learning, leading to inefficient training. Prioritized experience replay, however, assigns a priority (typically based on TD error) to each experience sample, making samples with higher TD errors more likely to be sampled. For each experience $\left(s_{t},a_{t},r_{t},s_{t+1}\right)$, its priority $p_{t}$ is calculated as follows:

(24) $$p_{t}=\left|\delta_{t}\right|+\varepsilon$$

where $\delta_{t}$ is the TD error of the experience, and ε is a small constant (to prevent the priority from being zero).

Among them

(25) $$\delta_{t}=r_{t}+\gamma \cdot Q^{\prime }(s_{t+1},a_{t+1};\theta^{\prime })-Q(s_{t},a_{t};\theta )$$

where $r_{t}$ is the reward, γ is the discount factor, $Q^{\prime }(s_{t+1},a_{t+1};\theta^{\prime })$ is the target *Q* value, and $Q(s_{t},a_{t};\theta )$ is the current *Q* value.

Therefore, in the prioritized experience replay, the sampling probability p(i) of the sample is

(26) $$p(i)=\frac{p_{i}^{\alpha }}{\sum_{j}p_{j}^{\alpha }}$$

where α is an adjustable hyperparameter that controls the degree of priority for experience playback.

Traditional *Q*-learning methods suffer from training instability due to overestimation of the *Q*-value, especially in complex reinforcement learning tasks. This overestimation problem stems from the fact that the calculation of the target value relies on the same *Q*-network, leading the agent to make suboptimal decisions. Dual *Q*-learning, by introducing two independent *Q*-networks (critic1 and critic2) for action selection and action evaluation, respectively, reduces the overestimation of the *Q*-value. Specifically, in standard *Q*-learning, the target *Q*-value $y_{t}$ is calculated using a single *Q*-network:

(27) $$y_{t}=r_{t}+\gamma \cdot Q(s_{t+1},a_{t+1};\theta^{\prime })$$

In dual *Q*-learning, two independent *Q*-networks, critic1 and critic2, are used. The specific calculations are shown below:

(28) $$y_{t}^{\mathrm{critic}1}=r_{t}+\gamma \cdot Q_{2}(s_{t+1},a_{t+1};{\theta^{\prime }}_{2})$$

(29) $$y_{t}^{\mathrm{critic}2}=r_{t}+\gamma \cdot Q_{1}(s_{t+1},a_{t+1};{\theta^{\prime }}_{1})$$

where $Q_{1}(s_{t+1},a_{t+1};{\theta^{\prime }}_{1})$ is the target estimate of the critic1 network, and $Q_{2}(s_{t+1},a_{t+1};{\theta^{\prime }}_{2})$ is the target estimate of the critic2 network.

The above calculations effectively avoid the overestimation of *Q*-values that occurs in traditional *Q*-learning during training. The updated formula is then derived as follows:

(30) $$\theta_{1}\leftarrow \theta_{1}+\alpha \cdot \nabla_{\theta_{1}}\left[{\left(y_{t}^{\mathrm{critic}1}-Q_{1}(s_{t},a_{t};\theta_{1})\right)}^{2}\right]$$

(31) $$\theta_{2}\leftarrow \theta_{2}+\alpha \cdot \nabla_{\theta_{2}}\left[{\left(y_{t}^{\mathrm{critic}2}-Q_{2}(s_{t},a_{t};\theta_{2})\right)}^{2}\right]$$

where α is the learning rate, which is used to control the step size of each update.

Therefore, this study introduces prioritized experience replay and dual *Q*-learning on the basis of DDPG. Assuming that is the parameter of the current policy network (Actor), then $\phi_{1}$ and $\phi_{2}$ are the parameters of two Critic networks. Then, one of the Critic networks in dual *Q*-learning can be used to evaluate it:

(32) $$\theta \leftarrow \theta +\alpha \cdot \nabla_{\theta }E\left[Q_{1}(s_{t},\pi (s_{t};\theta );\phi_{1})\right]$$

The Critic network is still updated based on the minimum mean square error of the *Q* value, but two independent Critic networks are used to reduce the bias of overestimating the *Q* value:

(33) $$\phi_{1}\leftarrow \phi_{1}+\alpha \cdot \nabla_{\phi_{1}}E\left[{\left(y_{t}^{\mathrm{critic}1}-Q_{1}(s_{t},a_{t};\phi_{1})\right)}^{2}\right]$$

(34) $$\phi_{2}\leftarrow \phi_{2}+\alpha \cdot \nabla_{\phi_{2}}E\left[{\left(y_{t}^{\mathrm{critic}2}-Q_{2}(s_{t},a_{t};\phi_{2})\right)}^{2}\right]$$

**Figure 5:**
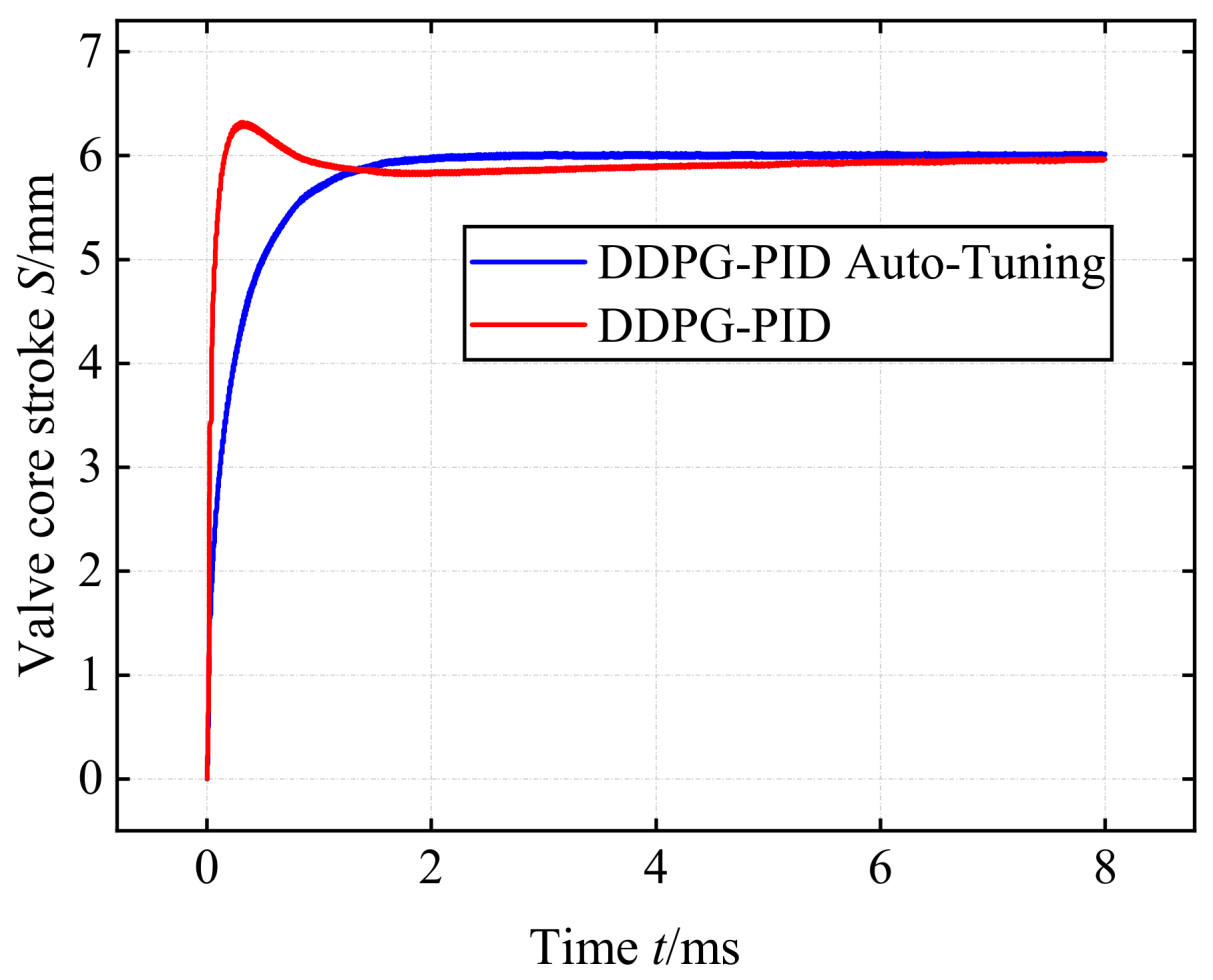
Different control strategies affect the valve core stroke control results.

To provide an initial comparison, the proposed controller was evaluated against the baseline DDPG-PID controller under the same simulation conditions. The baseline employs uniform experience replay and a single Critic network, whereas the proposed configuration incorporates prioritized experience replay and two Critics. Figure 5 presents the corresponding valve-spool responses for the tested operating condition.

As can be seen from the figure, DDPG-PID Auto-Tuning exhibits a significant advantage in response. Firstly, DDPG-PID Auto-Tuning responds more smoothly in the initial stage and quickly approaches the target valve spool position (6 mm) within a short time. In contrast, DDPG-PID shows a large overshoot in the initial stage and converges to the target position more slowly, exhibiting prolonged oscillations. More importantly, DDPG-PID Auto-Tuning stabilizes near the target position earlier and remains within a small error range, demonstrating better stability and accuracy. This indicates that the improved control strategy effectively avoids the oscillation and overshoot problems common in traditional PID controllers, improving the system’s control performance and response speed.

## 4. Case Study

The following sections will use simulation systems and experimental data to verify the practicality of the proposed method. To further demonstrate the effectiveness of the method, we will analyze the control results for different valve core strokes.

### 4.1. Study Subjects

**Figure 6:**
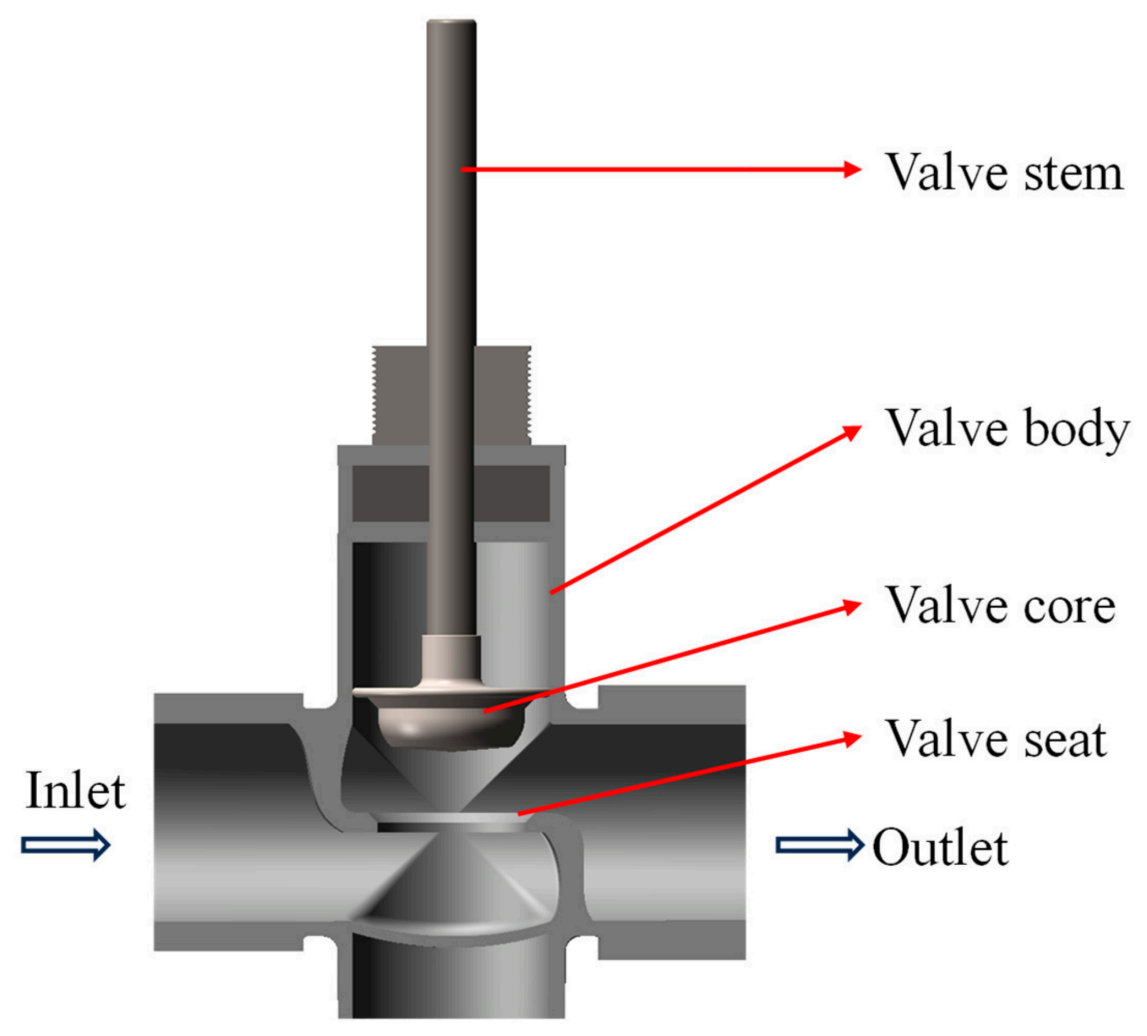
Schematic diagram of 3D model of control valve.

**Figure 7:**
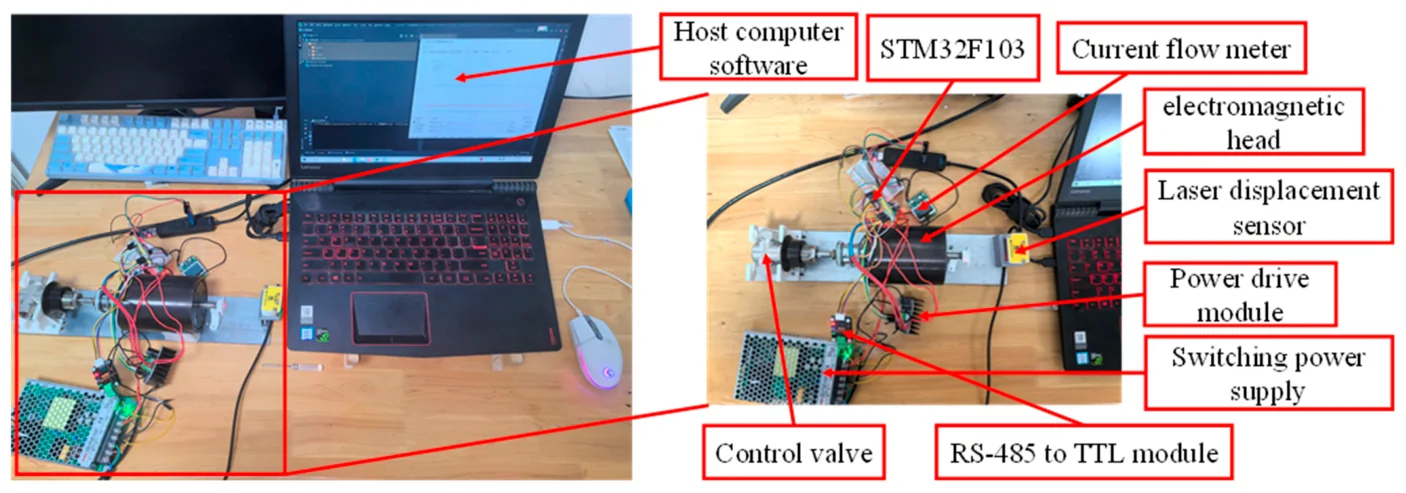
The overall structure of the control system.

The controlled valve investigated in this study is the laboratory-developed prototype shown in Figure 6, with a nominal diameter of DN20 and a nominal pressure designation of PN25. Because it is a custom-built prototype, it has no commercial model designation. Figure 6 presents the valve assembly, which mainly consists of the valve stem, valve body, valve spool, and valve seat. During operation, the electromagnetic linear actuator shown in Figure 7 drives the valve spool through the valve stem. The tested spool-stroke range is 0–20 mm, and the inlet and outlet flow directions are indicated by arrows in Figure 6.

Figure 7 shows the components of the entire control system.

This diagram illustrates a system comprising several key components that work together to achieve precise control of an electromagnetically driven control valve. The left side shows the connection between the host computer and the STM32F103 microcontroller (Jinhua Qianjian Technology Co., Ltd., Jinhua, China), which handles data processing, system control, and the real-time execution of the PID control algorithm. The PID controller deployed on the STM32 experimental platform is implemented using a discrete position method, with a control cycle of $T_{s}=10$ ms, corresponding to a control update frequency of 100 Hz. To suppress integral saturation, the integral state is limited to the range [−300, 300], and the PID correction output is also limited to the range [−300, 300]. The system also includes a current sensor, a laser displacement sensor, and a solenoid valve, used to monitor current changes, measure valve spool position, and control fluid flow, respectively. An RS-485 to TTL converter handles data communication, while a switching power supply module provides stable power. The right side shows the specific layout of these hardware components, which are connected by wiring to form a complete control platform that can perform real-time control and performance adjustment under complex operating conditions, demonstrating strong adaptability. Specifically, within the actual operating range of the valve spool, zero-point correction and local calibration are employed to compensate for the systematic linearity error of the displacement sensor. Following compensation, measurement uncertainty stems primarily from the resolution of 0.01 mm and the repeatability of 0.03 mm. Assuming both follow a rectangular distribution, the combined standard uncertainty is 0.0176 mm; with a coverage factor of *k* = 2, the expanded uncertainty is approximately 0.04 mm.

### 4.2. DDPG-PID Auto-Tuning Control Strategy Simulation System

**Figure 8:**
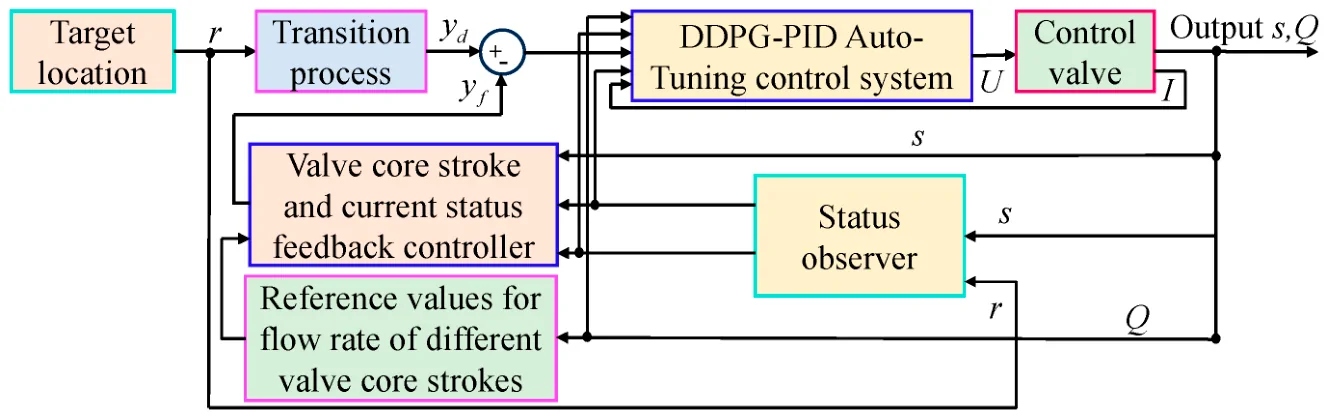
Simulation model structure diagram.

To further verify the feasibility of the proposed method, this section will utilize MATLAB/Simulink R2023b to build a simulation model and construct an experimental setup. By introducing the proposed DDPG-PID Auto-Tuning control strategy into the development board for system control, this study first establishes a simulation environment. The simulation results of different control strategies are compared to evaluate the performance of the DDPG-PID strategy in terms of dynamic response, stability, and robustness. Subsequently, the simulation results are applied to a practical hardware platform, and the advantages of the DDPG-PID strategy are further verified by comparing it with traditional PID and fuzzy + PID controllers. The hardware experimental setup includes sensors, actuators, and an embedded development board. Closed-loop control experiments are conducted on the system through real-time data acquisition and analysis. Figure 8 shows the block diagram of the simulation model built on the MATLAB/Simulink platform.

As shown in the diagram, the system takes the target position *r* as input, generates a reference quantity $y_{d}$ through a “transition process,” and forms a control error signal with the feedback $y_{f}$ at the addition/subtraction node. This error signal, along with the system state information, is sent to the DDPG-PID Auto-Tuning control system. This module, acting as the core controller, adaptively tunes the PID parameters online based on the current operating state and outputs a control quantity *U* to drive the subsequent “control valve,” thereby obtaining the system output displacement *s* and flow rate *Q*. Simultaneously, the output signal is fed back through feedback: on one hand, it enters the “state observer” to reconstruct/estimate the state *s*, providing a more accurate state input for the reinforcement learning controller; on the other hand, it enters the “valve spool stroke and current state feedback controller” and the “different valve spool stroke flow rate reference value” module, achieving matching and compensation between the electromagnetic linear actuator state and the reference quantity, ensuring the system’s tracking accuracy and stability under different operating conditions.

**Figure 9:**
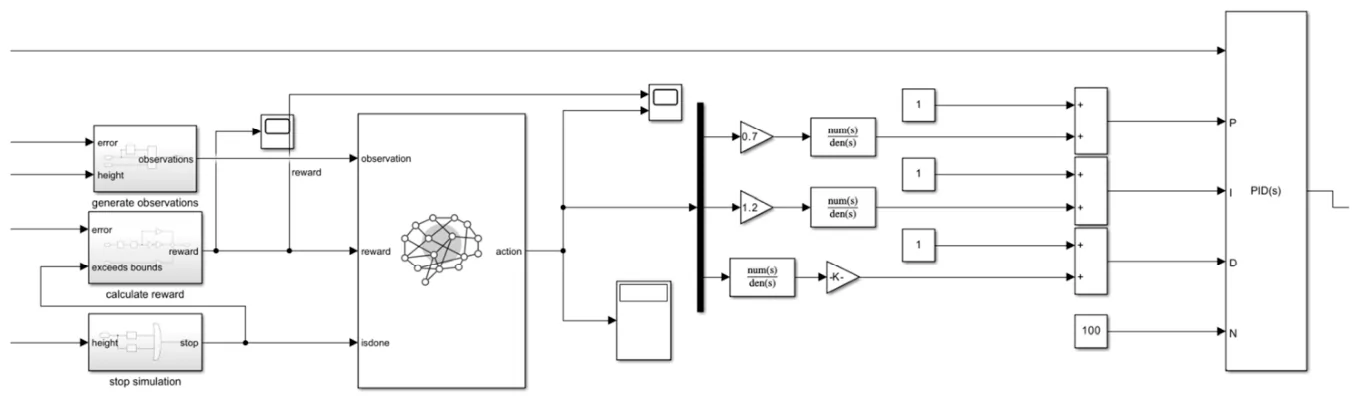
DDPG-PID Auto-Tuning Control Strategy System.

Figure 9 shows a partial schematic diagram of the control strategy system built in Simulink.

The diagram illustrates the entire reinforcement learning process. First, the system generates observation information by calculating the current error and valve spool stroke, using this information as input to the agent. Then, the environment calculates a reward based on the current state information and evaluates the effectiveness of the agent’s control actions using a reward function. Upon receiving the reward, the reinforcement learning agent generates control actions (i.e., PID parameters) based on the current observations. These control actions are then passed to the PID controller to adjust the actuator output, thereby controlling the behavior of the control valve. Furthermore, the control module in the diagram sets a condition to stop the simulation; when the current valve position flow exceeds a preset threshold, the system automatically terminates training. This structure allows the agent to optimize its control strategy in each round, enabling the PID parameters to gradually self-tune during simulation, achieving better control performance.

Based on Figure 8 and Figure 9, the closed-loop transfer function of the inner current loop can be further determined as follows:

(35) $$T_{i}(s)=\frac{C_{i}(s)G_{i}(s)}{1+C_{i}(s)G_{i}(s)}$$

Among them

(36) $$C_{i}(s)=K_{pi}+\frac{K_{i}}{s}+K_{d}s$$

(37) $$G_{i}(s)=\frac{I(s)}{PWM(s)}\approx \frac{K_{PWM}}{Ls+R}$$

where *L* is the inductance of the electromagnet coil, *R* is the coil resistance, and $K_{PWM}$ is the voltage gain of the driver.

The further system-wide (outer loop) closed-loop transfer function is

(38) $$T_{x}(s)=\frac{X_{actual}(s)}{X_{t\arg et}(s)}=\frac{C_{p}(s)\cdot T_{i}(s)\cdot G_{x}(s)}{1+C_{p}(s)\cdot T_{i}(s)\cdot G_{x}(s)}$$

Among them

(39) $$C_{p}(s)=K_{pp}+\frac{K_{ip}}{s}+K_{dp}s$$

(40) $$G_{x}(s)=\frac{X(s)}{I(s)}\approx \frac{K_{f}}{ms^{2}+cs+k_{eq}}$$

where $K_{f}$ is the electromagnetic force coefficient, *m* is the valve core mass, *c* is the damping coefficient, and $k_{eq}$ is the equivalent spring stiffness.

When the inner loop response is fast enough ($T_{i}(s)\approx 1$), it can be simplified to

(41) $$T_{x}(s)\approx \frac{C_{p}(s)\cdot G_{x}(s)}{1+C_{p}(s)\cdot G_{x}(s)}$$

**Figure 10:**
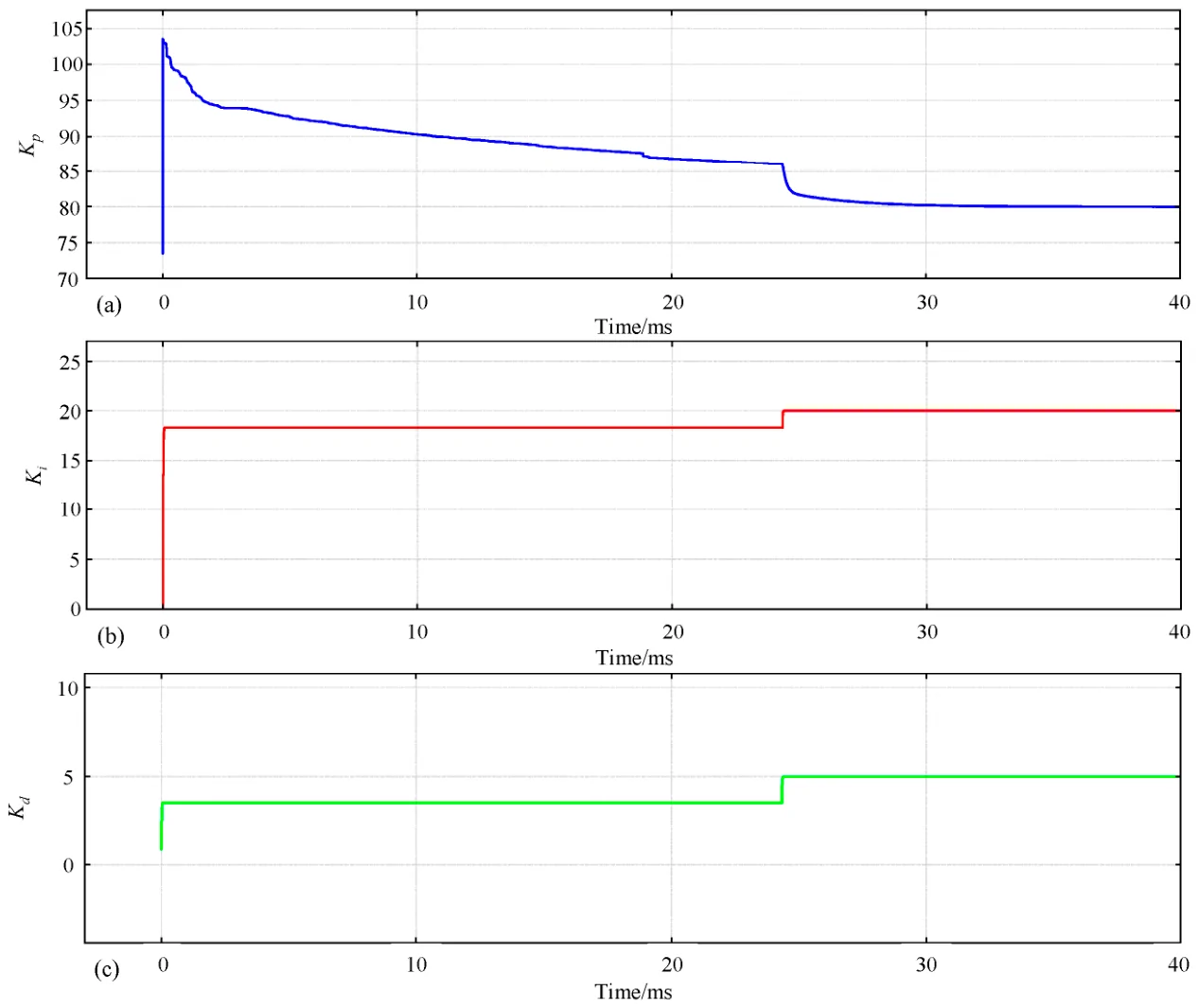
PID gain change process.

Figure 10 shows the gain variation in the PID during the experiment.

The figures illustrate the variation patterns of the three gain parameters of the PID controller during the experiment. It can be observed that the proportional gain, integral gain, and derivative gain are all rapidly set in the initial stage and adjusted according to the system response during subsequent control. In Figure 10a, the proportional gain shows a significant initial jump, then gradually decreases from approximately 103, indicating that the controller uses a large proportional action in the early stages of the response to increase the adjustment speed. As the system gradually stabilizes, the proportional gain continuously decreases, then drops again by a step around 25 ms, and eventually stabilizes at approximately 80 to reduce oscillations or overshoot caused by excessive proportional action. In Figure 10b, the integral gain rises rapidly to approximately 18.3 in the initial stage and remains constant for a relatively long time, then increases by a step to 20 at approximately 25 ms. This indicates that the controller enhances the integral action after the system enters a relatively stable phase to further reduce steady-state error. In Figure 10c, the derivative gain initially rises to approximately 3.5, then increases to 5 at approximately 25 ms and remains stable. This change illustrates that the controller improves the system’s dynamic performance, suppresses fluctuations in the response process, and enhances the stability of the control process by enhancing the derivative action. Therefore, the figure reflects that the PID parameters are not fixed during the experiment, but are adjusted in stages according to the system’s operating state, initially focusing on rapid response, and later enhancing steady-state accuracy and dynamic stability by reducing the proportional gain and increasing the integral and derivative gains.

**Figure 11:**
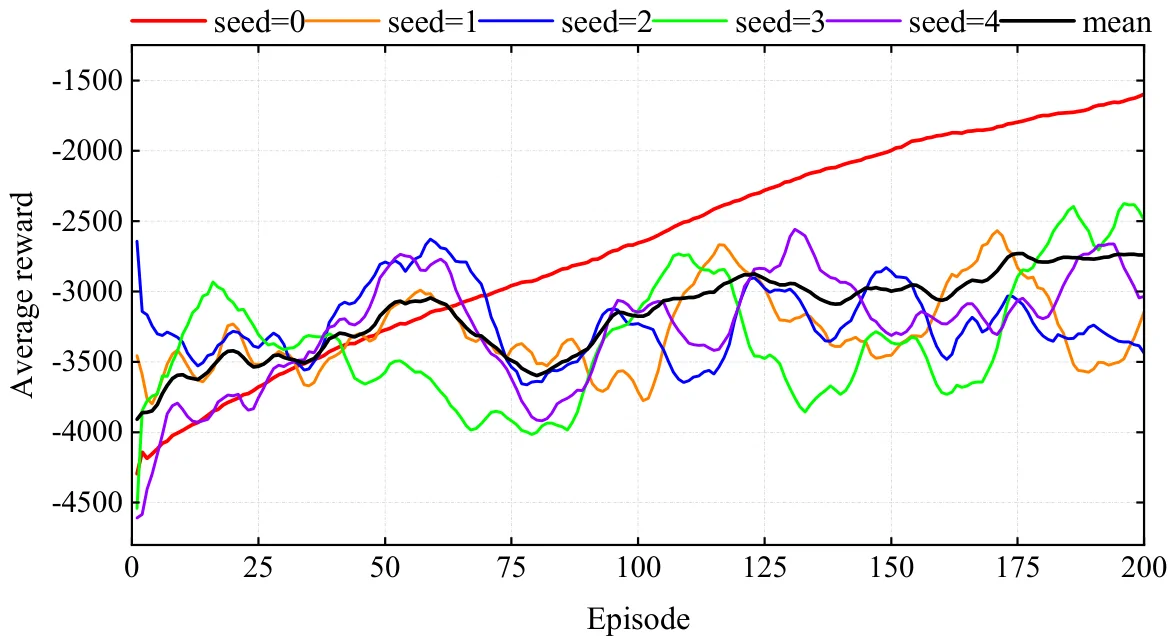
Average reward result under 5 random seeds.

Figure 11 shows the average reward results of the proposed method under five random seeds. The figure reveals significant differences in the curves for different random seeds. The colored curves represent the average reward changes in each independent training iteration, while the black curve represents the average across the five training iterations. Since a larger reward value (i.e., a smaller absolute value of a negative value) indicates better control, the overall increase in average reward suggests that the agent gradually improved its control strategy during training. Seed 0 showed the most continuous reward improvement, and its final reward was significantly higher than the final rewards of the other training runs. In contrast, Seeds 1–4 exhibited repeated fluctuations during training, and their final reward levels remained inconsistent even after 200 iterations. The dispersion between the different curves indicates that the training results are affected by the initial network weights, exploration noise, and the empirical sampling process.

Table 2 lists the hyperparameters used during the training process.

### 4.3. Simulation and Experimental Research

#### 4.3.1. Step Response at Different Target Locations

**Figure 12:**
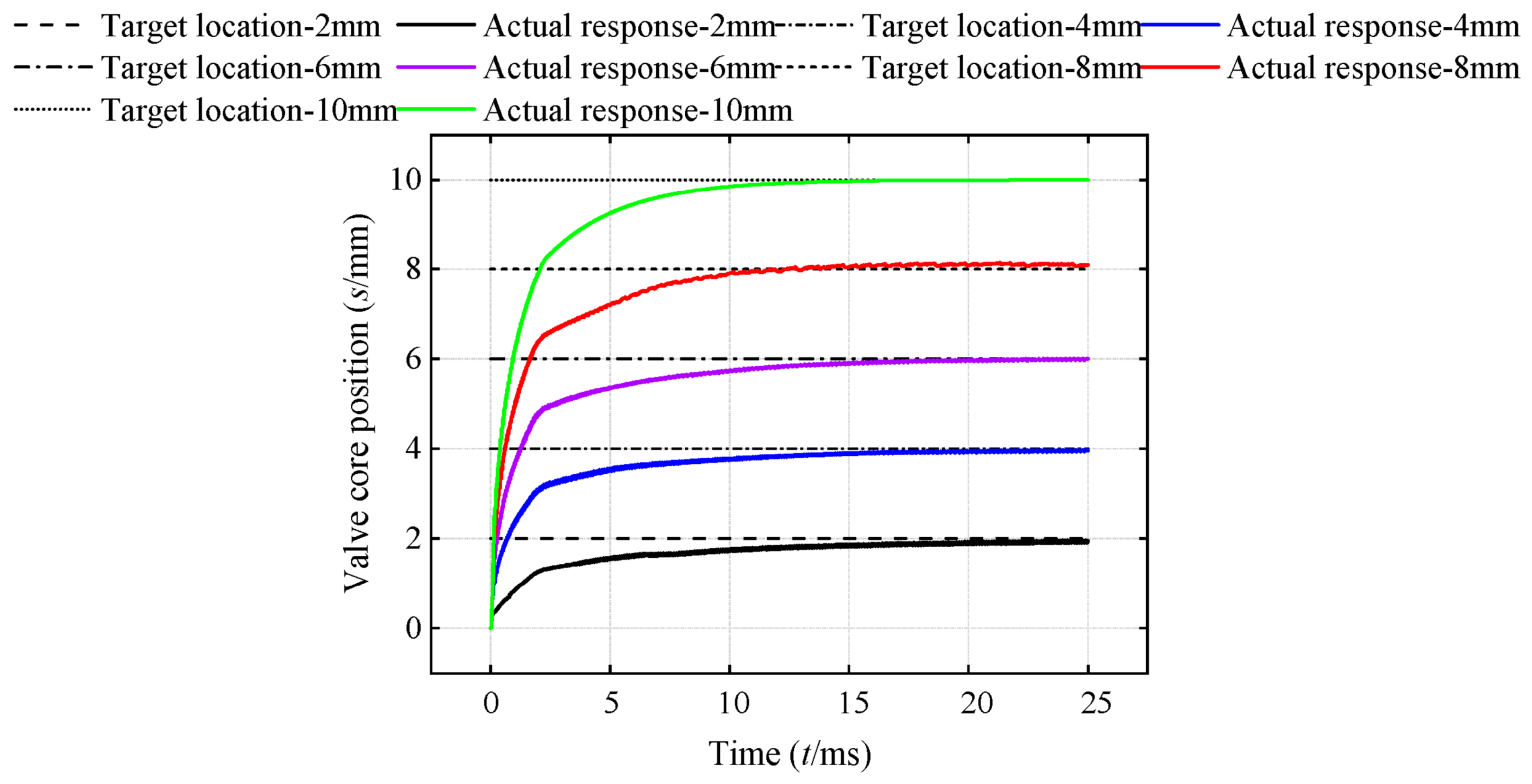
Step response with different valve core strokes.

To verify the adjustability of the control system of the control valve directly driven by the electromagnetic linear actuator under different valve spool strokes, this section analyzes the step response of the target stroke positions (2 mm, 4 mm, 6 mm, 8 mm, and 10 mm) using the DDPG-PID Auto-Tuning control strategy based on the simulation system. Figure 12 shows the step response results for different valve spool strokes without changing any control parameters.

As shown in the figure, the system’s response time and response amplitude increase with the increase in the target stroke of the valve core. For a target position of 2 mm, the system responds quickly and can reach and stabilize at the target position with relatively high accuracy. As the target position increases (6 mm, 8 mm, 10 mm), the system’s response time gradually increases, and the actual response value gradually approaches the target value. Particularly when the target position is 10 mm, the system’s response speed is the slowest, with a response time within 10 ms, but it eventually stabilizes near the target position, demonstrating that the system can adapt to changes in different target positions while maintaining high control accuracy. Based on the above, it can be found that the overall control accuracy error of the system is within ±0.04 mm.

#### 4.3.2. Simulation and Experiment in On/Off Valve Mode

When the control system of the control valve directly driven by the electromagnetic linear actuator is set to on/off valve mode, it can achieve timed opening and closing, and continuous switching within a variable period. In the following study, the preset value of the valve core stroke is 6 mm, and the period is 40 ms.

**Figure 13:**
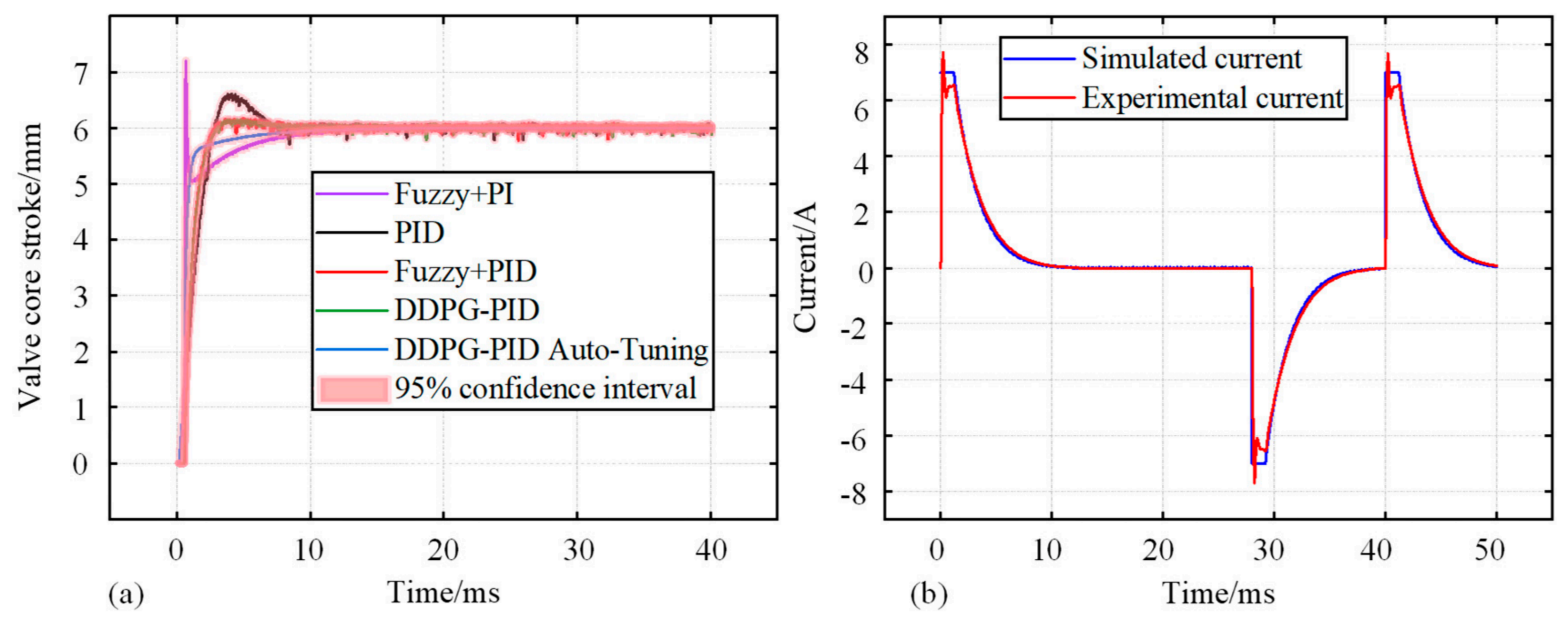
Simulation and experiment of fixed stroke. (**a**) Valve core stroke response under different control strategies. (**b**) Current response under fixed stroke.

To further verify the performance of the DDPG-PID Auto-Tuning control strategy, it was compared with traditional PID control, DDPG-PID control, fuzzy + PI control and fuzzy + PID control strategies. Experiments were conducted to analyze the performance of the five control strategies in terms of system dynamic response and current response. Among them, the Fuzzy + PID controller was employed only as a comparative method in this study. It adopts a two-input, three-output Mamdani-type fuzzy inference structure, in which the tracking error *E* and error variation *EC* are used as inputs, and the PID-gain correction terms $K_{p}$, $K_{i}$, and $K_{d}$ are generated to adjust the PID gains online. The universes of discourse of both inputs are [−6, 6]. The output universe of $K_{p}$ is [−3, 3], whereas those of $K_{i}$ and $K_{d}$ are both [−6, 6]. Seven triangular membership functions, namely NB, NM, NS, ZO, PS, PM, and PB, are assigned to each input and output variable. The rule base contains 147 rules organized into three 7×7 rule tables for adjusting $K_{p}$, $K_{i}$, and $K_{d}$, respectively. Mamdani inference is employed, with the minimum operator for AND implication, the maximum operator for OR aggregation, and the centroid method for defuzzification. Figure 13 shows the system response and current changes under different control strategies.

Figure 13 compares the experimental validation data under fixed-stroke operating conditions, with the light pink shaded areas representing the corresponding instantaneous 95% confidence intervals. Figure 13a presents the test results of spool displacement responses obtained using traditional PID, Fuzzy + PID, Fuzzy + PI, baseline DDPG-PID, and the DDPG-PID Auto-Tuning controller proposed in this paper, respectively. All five controllers eventually track the target displacement of 6 mm, although differences are observed in their transient and steady-state characteristics. The conventional PID controller produces the largest overshoot and the most pronounced transient oscillations. The fuzzy + PID controller reduces the overshoot and enters the prescribed settling band slightly earlier than the proposed controller, but it retains a greater steady-state displacement fluctuation amplitude. The baseline DDPG-PID controller provides improved damping and suppresses the transient oscillations more effectively than the conventional PID controller. The Fuzzy + PI controller produces the maximum overshoot during the initial stage, with the spool displacement briefly reaching approximately 7.2 mm before dropping significantly below the target value and gradually converging. The proposed DDPG-PID Auto-Tuning controller produces a smaller overshoot and the lowest peak-to-peak displacement variation after stabilization, resulting in a smoother steady-state response. Although its settling time is slightly longer than that of the fuzzy + PID controller, the difference is only a few milliseconds and is not considered practically significant for the control valve examined in this study. Therefore, the comparison reflects a trade-off between settling time and steady-state displacement fluctuation rather than an absolute advantage in all performance indices. Figure 13b compares the simulated and experimental current responses under the DDPG-PID Auto-Tuning strategy. The two curves are highly consistent during the positive, negative, and repeated current adjustment processes, with only slight deviations at transient peaks, and both quickly return to a stable state after each adjustment. The above results validate the reliability of the proposed method and demonstrate that the DDPG-PID Auto-Tuning approach achieves stable current regulation, precise spool stroke control, and superior overall control performance.

The specific calculation for the 95% confidence interval is as follows:

(42) $$CI_{95\%}(t)=\bar{x}(t)\pm t_{0.975,n-1}\frac{s(t)}{\sqrt{n}}$$

The lower and upper limits are

(43) $$CI_{\mathrm{lower}}(t)=\bar{x}(t)-t_{0.975,n-1}\frac{s(t)}{\sqrt{n}}$$

(44) $$CI_{\mathrm{upper}}(t)=\bar{x}(t)+t_{0.975,n-1}\frac{s(t)}{\sqrt{n}}$$

Among them

(45) $$s(t)=\sqrt{\frac{1}{n-1}\sum_{i=1}^{n}{\left[x_{i}(t)-\bar{x}(t)\right]}^{2}}$$

(46) $$\bar{x}(t)=\frac{1}{n}\sum_{i=1}^{n}x_{i}(t)$$

where $x_{i}(t)$ is the spool displacement at time *t* for the *i*-th independent repeated experiment, *n* is the number of experiments, *t* is the time point, $\bar{x}(t)$ is the average spool displacement across *n* experiments at time *t*, s(t) is the sample standard deviation of the *n* experiments, $CI_{\mathrm{lower}}(\cdot )$ is the lower limit of the 95% confidence interval, and $CI_{\mathrm{upper}}(\cdot )$ is the upper limit of the 95% confidence interval.

**Figure 14:**
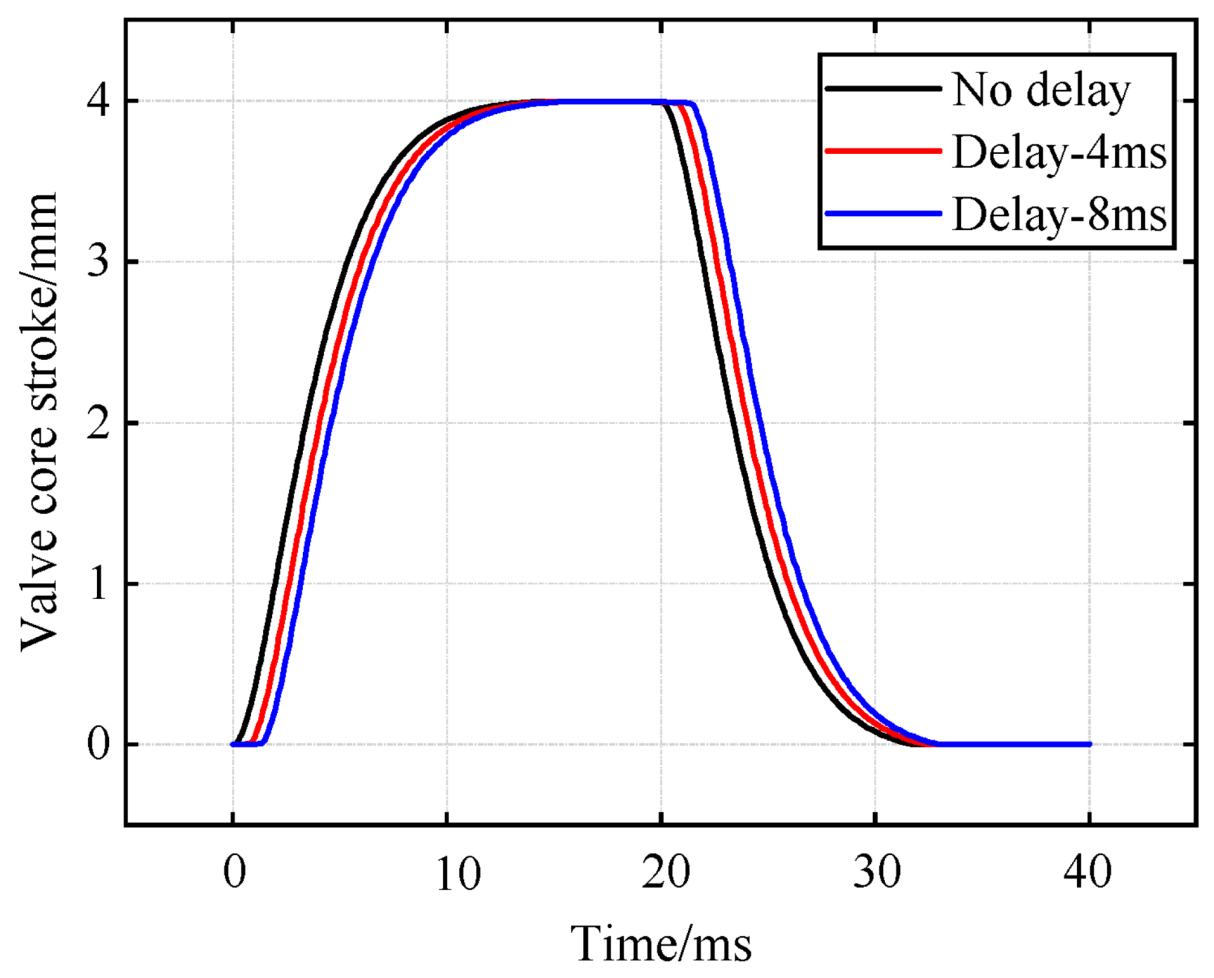
Simulation results at different timing points.

To evaluate the performance of the DDPG-PID Auto-Tuning control system in practical applications, especially under different control delay conditions, we conducted simulation tests at various delay times. The delay of a control system typically affects its performance, particularly in applications requiring fast response and precise tracking. Figure 14 shows the spool stroke response of the DDPG-PID Auto-Tuning control system under different delay conditions, including no delay, 4 ms delay, and 8 ms delay.

As can be observed from the graph, the system response trend remains consistent under no delay (black curve), 4 ms delay (red curve), and 8 ms delay (blue curve), and all three can accurately stabilize at the target position after final stabilization. The impact of delay on the system is mainly reflected in the initial stage of response. As the delay time increases, the rise time of the system is slightly prolonged, and the rise process of the response is slightly slower under 4 ms and 8 ms delays, but the overall performance is good, and the system can still maintain high control accuracy and stability.

To further verify the effectiveness of the DDPG-PID Auto-Tuning control system, Table 3 shows the error values of the valve core under different strokes. The results were obtained by calculating the average of five actual repeated experiments.

The table shows that as the valve spool stroke increases, both the maximum and minimum values also increase, indicating that the system’s output displacement range gradually expands. The range column displays the range of system position variation for each valve spool stroke; the range increases with the stroke. The error range column shows the error range of the control system under different valve spool strokes. The average measured displacements are close to the corresponding target displacements, with absolute deviations between the average measured values and the target values ranging from 0 to 0.018 mm. Furthermore, the standard deviation of the results from five repeated measurements ranges from 0.005 to 0.032 mm, indicating relatively small fluctuations in results across different experiments under the tested conditions. The overall error range remains within ±0.04 mm, further verifying that the system maintains high control accuracy and small error under various valve spool strokes, demonstrating strong stability and good precision control.

#### 4.3.3. Ablation Experiment

**Figure 15:**
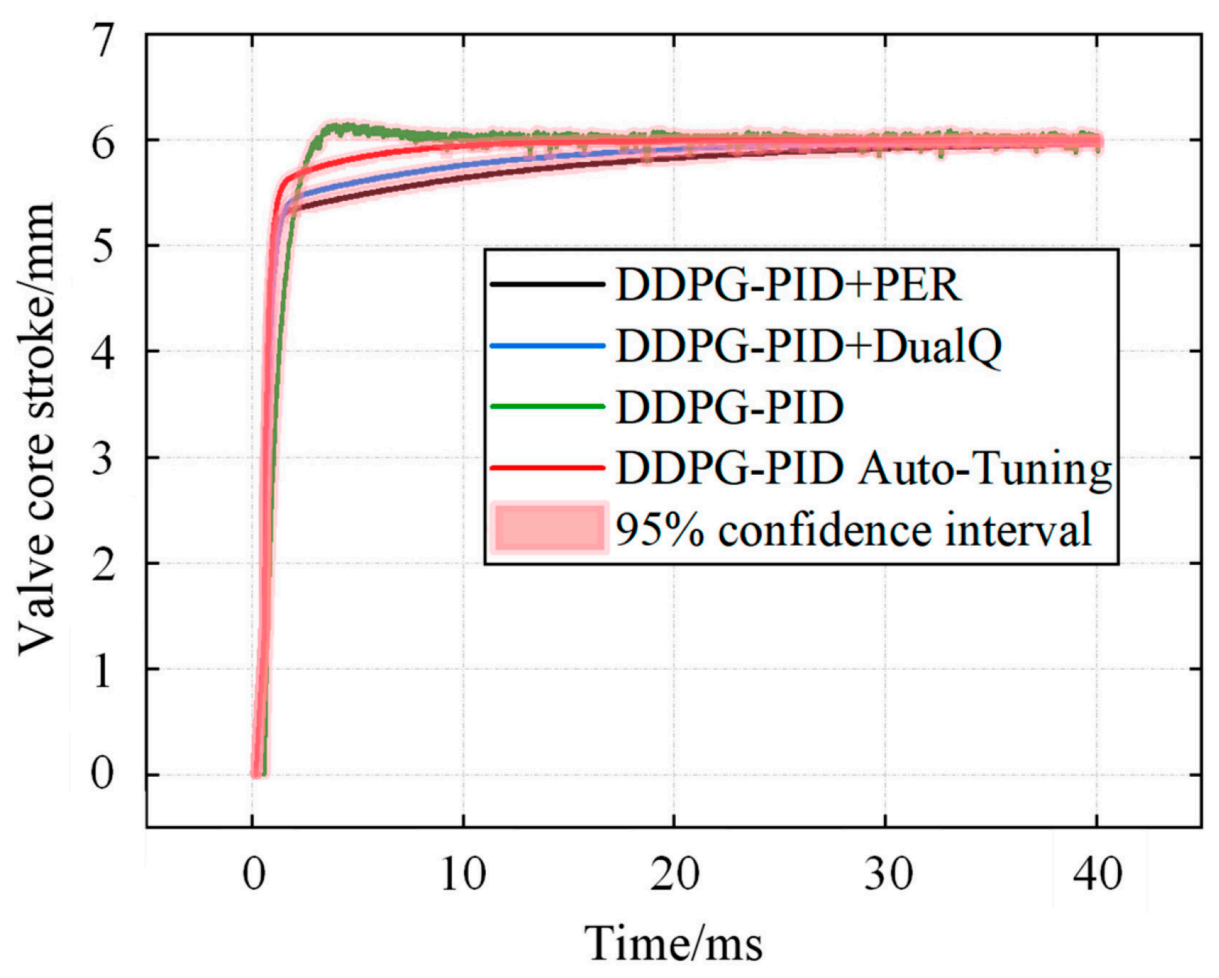
Ablation test results.

To further validate the effectiveness of the DDPG-PID Auto-Tuning method, we added ablation experiments with prioritized experience replay disabled, double Q learning, and standard DDPG-PID to quantify the contributions of the two improved modules. Figure 15 illustrates the relevant results.

As shown in the figure, all four methods eventually converge to the vicinity of the target value, but their dynamic response processes differ significantly. The shaded regions in the figure represent the corresponding pointwise 95% confidence intervals. The standard DDPG-PID method exhibits significant overshoot after the initial rise and some fluctuations near the steady state, indicating that the original DDPG-PID controller is still insufficient in suppressing transient oscillations. Introducing prioritized experience replay reduces overshoot and improves response stability, but its convergence speed is relatively slow. The DDPG-PID + DualQ method also effectively suppresses overshoot and exhibits a smoother response process, indicating that dual Q-learning helps improve training stability and reduce the impact of Q-value overestimation. Compared to methods that introduce only one improvement module, the complete DDPG-PID Auto-Tuning method has a faster response speed, smaller overshoot, and a smoother convergence process, converging more stably to the vicinity of the 6 mm target displacement. Therefore, both prioritized experience replay and dual Q-learning have a positive effect on improving control performance, and their combination can effectively improve the overall control effect.

#### 4.3.4. Simulation and Experiment in Servo Valve Mode

**Figure 16:**
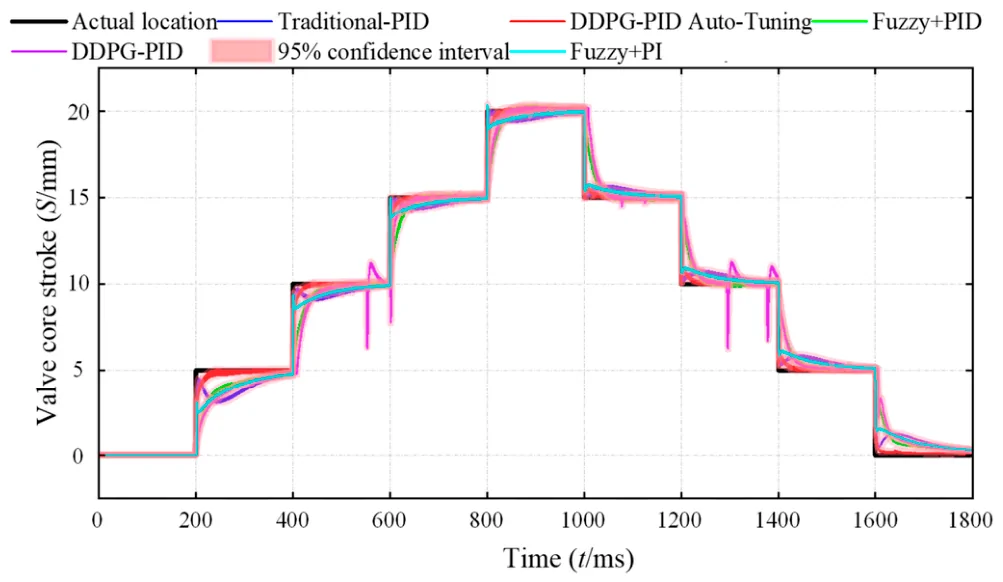
Comparison of results of different control strategies in servo valve mode.

To further evaluate the performance of different control strategies in practical applications, Figure 16 shows a comparison of control results using traditional PID, fuzzy + PID, DDPG-PID, fuzzy + PI, and DDPG-PID Auto-Tuning control strategies in servo valve mode.

The figure shows the servo adjustment process at different target preset valve spool strokes of 25%, 50%, 75%, and 100%. The black curve denotes the reference position, while the blue, red, green, light blue, and purple curves represent the responses of the traditional PID, DDPG-PID Auto-Tuning, fuzzy + PID, fuzzy + PI, and baseline DDPG-PID controllers, respectively. The pink shaded areas indicate the instantaneous 95% confidence intervals for the different control methods. The figure shows that at each step change, DDPG-PID Auto-Tuning exhibits the fastest response speed and the smallest overshoot, with almost no system oscillation, and can quickly stabilize at the target position. In contrast, traditional PID exhibits significant overshoot and oscillation after each step change and has a longer settling time. While fuzzy + PID and fuzzy + PI improve overshoot and oscillation, they still fail to achieve the control accuracy and stability of DDPG-PID Auto-Tuning. The baseline DDPG-PID controller generally tracks the reference command but produces noticeable transient spikes and oscillations around several step transitions. Furthermore, the five strategies in the figure show similar trends, indicating that the DDPG-PID Auto-Tuning control strategy not only provides a fast response but also effectively reduces oscillation, significantly improving the system’s stability and accuracy.

#### 4.3.5. Valve Core Stroke 6 mm Experimental Results

**Figure 17:**
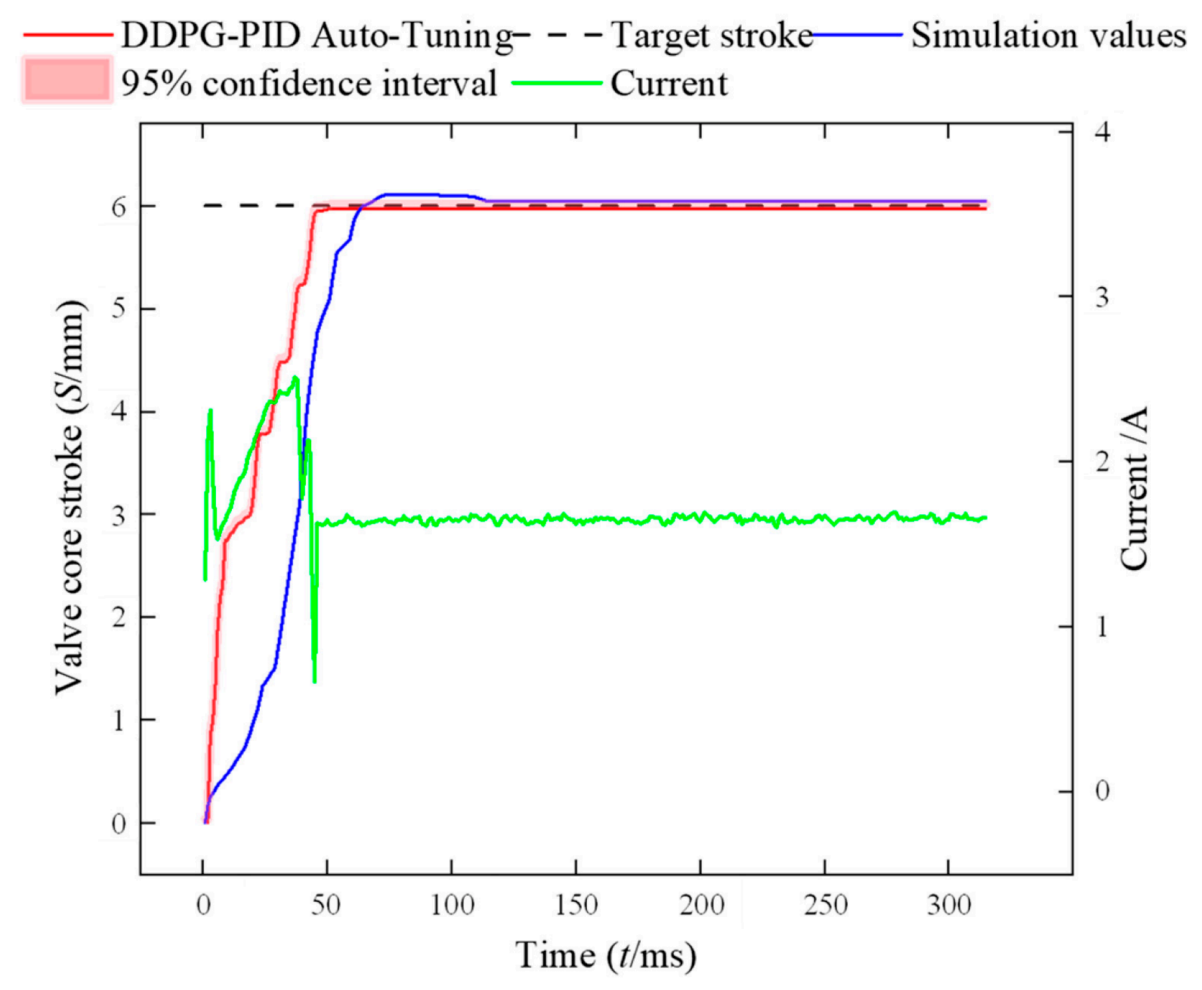
Response and current at a target stroke of 6 mm.

Figure 17 shows the comparison between experimental and simulation results when the valve core stroke is set to 6 mm. The red curve in the figure represents the measured valve core stroke, while the light red shaded area indicates the 95% confidence interval for the corresponding stroke. It can be seen that as the control process progresses, the actual displacement of the valve core gradually approaches and eventually stabilizes near the target stroke of 6 mm, indicating that the constructed control system can effectively drive the valve core to move towards the target position and maintain good steady-state characteristics near the target position. The black dashed line represents the target stroke of 6 mm. The green curve is the drive current signal, which fluctuates somewhat in the initial stage of adjustment according to the actuator’s movement requirements. It gradually stabilizes after the valve core approaches the target position, indicating that the drive current can adapt well to the actuator’s dynamic adjustment requirements, and the current control process has good stability. The blue curve represents the simulation system output result, showing that the simulation model also achieves good trajectory tracking and steady-state control effects. Therefore, during the entire adjustment process, the actual stroke stability value is 5.97 mm, with a deviation of −0.03 mm from the target value, while the simulated stroke stability value is 6.04 mm, with a deviation of 0.04 mm from the target value; simultaneously, the system current eventually stabilizes at a low level. The above results show that the adopted DDPG-PID Auto-Tuning control method can effectively improve the dynamic response capability and steady-state control accuracy of the actuator, realize high-precision adjustment of valve core displacement, and ensure the stability of the system operation process.

#### 4.3.6. Experimental Results for Arbitrary Stroke of the Valve Core

**Figure 18:**
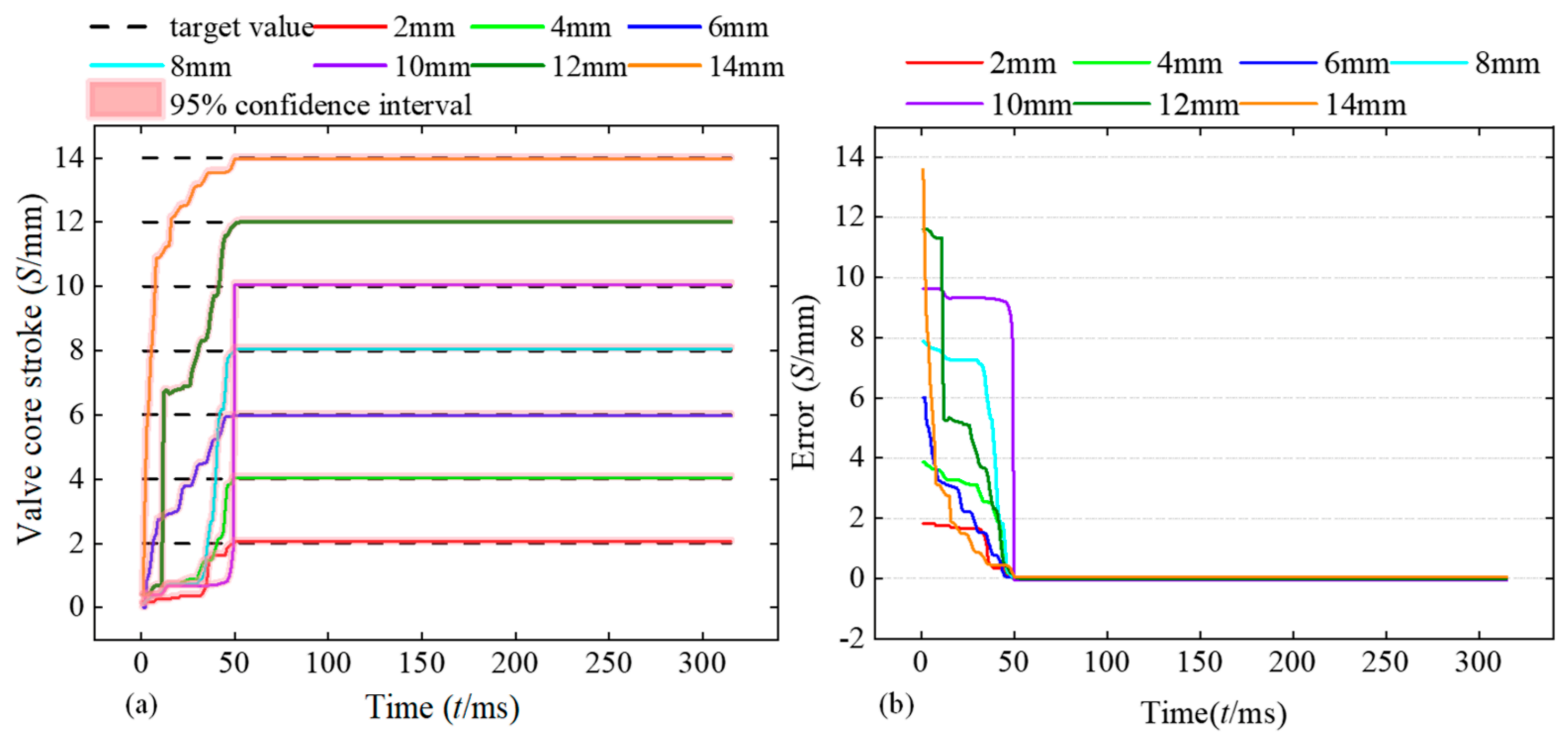
Experimental results and errors. (**a**) Test results under different valve core strokes. (**b**) Error results at different valve spool strokes.

To verify the designed control system’s ability to track different target displacement commands and its adaptability across the entire stroke range, arbitrary stroke tracking experiments were conducted at several typical target stroke points of the actuator. The target values set for the experiments were 2 mm, 4 mm, 6 mm, 8 mm, 10 mm, 12 mm, and 14 mm. For each target stroke, the response curve of the actuator’s output displacement over time was recorded, and the corresponding 95% confidence interval was calculated. The experimental results are shown in Figure 18. As shown in the figure, under different target input conditions, the actuator was able to start from its initial position, complete dynamic tracking of the set stroke in a short time, and eventually stabilize near the corresponding target value. This indicates that the constructed control system has good arbitrary stroke adjustment capability and strong target tracking performance.

From the perspective of dynamic response, the system exhibits fast start-up characteristics under different stroke commands. The actuator can quickly establish a displacement response after the input control signal, indicating that the system has good driving capability and control sensitivity. As shown in Figure 18a, for smaller target strokes, such as 2 mm and 4 mm, the actuator can achieve stable tracking quickly, with a smooth response and small overshoot. For medium target strokes, such as 6 mm, 8 mm, and 10 mm, the displacement response curve maintains a good upward trend overall. Although there is some adjustment transition in local stages, it can enter a steady state in a short time. For larger target strokes, such as 12 mm and 14 mm, the actuator can still achieve effective tracking and eventually stabilize near the target value, indicating that the control method still has good adaptability and control capability under larger stroke conditions. As shown in Figure 18b, the initial error increases with the target stroke and gradually decreases as the valve spool approaches the target position. Although step-like changes occur during the transient phase under certain operating conditions, the errors corresponding to each stroke ultimately converge to near-zero values, with no significant error fluctuations observed after stabilization. Based on the above, it can be found that as the target stroke increases, the system response time increases, but this change is consistent with the objective law that the actuator needs to overcome longer motion distances and more complex nonlinear factors under larger displacement output conditions. Especially when the stroke is 14 mm, the experimental test result (13.97 mm) is slightly lower than the target stroke, with a deviation of −0.03 mm from the target value. This may be related to factors such as the accumulated frictional resistance, hysteresis effect and enhanced spring reaction force after the movement distance increases under large stroke conditions. However, the deviation is very small and does not affect the overall control accuracy and stability of the system.

**Figure 19:**
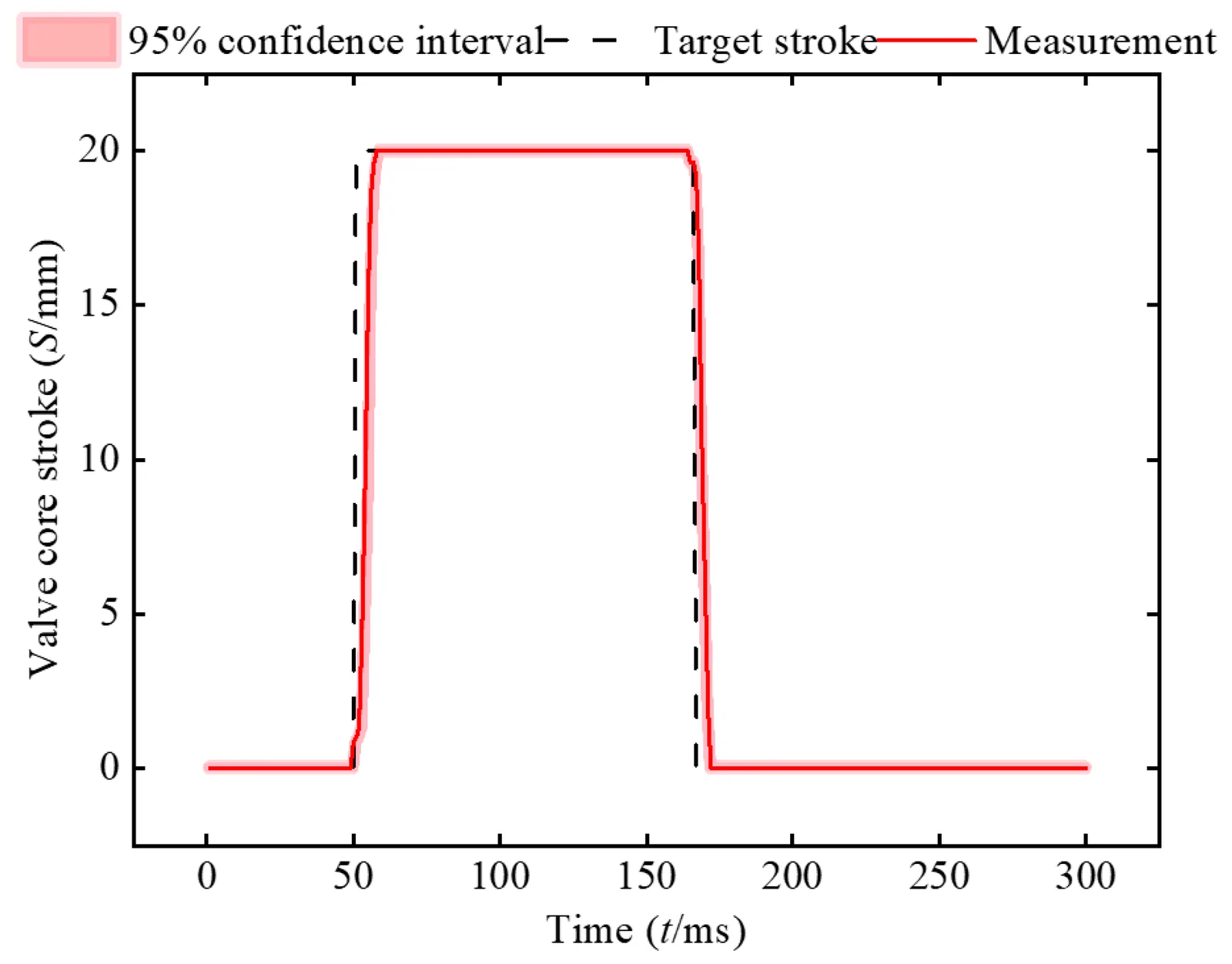
Test results in on/off valve mode.

To further verify the motion response capability and position holding performance of the proposed control method in the on/off valve mode, the test results in the on/off valve mode are shown in Figure 19. The test was conducted under a switching control condition with a target stroke of 20 mm. In the figure, the dashed line represents the target stroke, the solid red line represents the actual displacement response of the actuator, and the pink area represents the 95% confidence interval. Comparing the relationship between the two over time can intuitively reflect the dynamic response characteristics of the actuator and its ability to track target commands during the opening and closing processes.

As shown in the figure, the valve core remains closed (0 mm) in the initial stage, and the system operates relatively stably. When an opening command is input at 50 ms, the actual displacement of the actuator quickly rises from 0 mm to the fully open state (20 mm) and remains stable at the target position, indicating that the system has a fast response speed and good position holding capability during the opening process. Subsequently, when a closing command is input at approximately 165 ms, the actual displacement of the valve core quickly falls back from 20 mm to 0 mm, and the return process is relatively smooth, without obvious oscillation or large overshoot. The 95% confidence intervals shown in the figure are generally narrow and closely clustered around the measured response curve across the activation, holding, and deactivation phases, indicating good consistency and repeatability in the responses observed across the five independent experiments. Therefore, the actual displacement curve is basically consistent with the trend of the target stroke command, indicating that the designed control system can realize the rapid opening and closing of the valve core under large stroke requirements in the on/off valve mode, and has good action accuracy, timely response, and operational stability.

## 5. Conclusions

This study addresses the challenges of strong nonlinearity, parameter uncertainty, frequent changes in operating conditions, and reliance on experience for traditional PID parameter tuning in electromagnetic linear actuator-driven control valves during on/off and servo control. A reinforcement learning control method based on DDPG for PID parameter self-tuning is proposed, and its control strategy is designed, simulated, and experimentally verified. Results show that the constructed control system can adapt to both on/off and servo control requirements. The proposed reinforcement-learning control strategy establishes a mapping from the measured system states to the PID gains, enabling the gains to be adaptively adjusted according to the current operating state. Although an approximate valve model is required to construct the offline Simulink training environment, the deployed controller does not require the model equations to be solved online. Furthermore, the introduction of an experience playback mechanism and a soft update strategy for the target network effectively improves sample utilization efficiency and enhances the stability and convergence performance of the training process. Simulation and experimental results further demonstrate that the proposed method can stably achieve timed opening, holding, and closing actions in on/off valve mode, and in servo valve mode, it can achieve precise control of the preset target position within a valve core stroke range of 0~20 mm, with a positioning accuracy error of 0.04 mm and a response time of less than 50 ms. Compared with traditional PID, DDPG-PID, fuzzy + PI, and fuzzy + PID methods, the overall control performance of this DDPG-PID auto-tuning strategy is superior to all four methods in terms of tracking capability and system overshoot. Therefore, the DDPG-PID auto-tuning strategy can effectively improve the dynamic response performance, control accuracy, and system control robustness of electromagnetic linear actuator-driven control valves.

The proposed DDPG-PID Auto-Tuning strategy has currently been validated only on the control valve investigated in this study, and its applicability to other valve types and operating conditions requires further verification. Although the strategy establishes an adaptive mapping between the system states and PID gains, the computational cost of the Actor network may limit its application to systems with faster dynamics. Therefore, its cross-object generalization and real-time performance cannot yet be broadly determined. Future research will evaluate the strategy under different valve sizes, actuator configurations, more demanding control scenarios, power-supply conditions, and control periods.

## Figures and Tables

**Table 1:** Model reward function weights.

Symbols	Numerical Value	Function
$\omega_{e}$	5	Tracking error penalty
$\omega_{\Delta e}$	2	Penalty for error variation
$\omega_{u}$	0.1	Control input penalty
$\omega_{K}$	0.1	PID gain change penalty
$\omega_{o}$	40	Overshoot penalty
$r_{b}$	15	Reward for entering the allowable error range
ε	0.08 mm	Permissible displacement error

**Table 2:** Model training hyperparameters.

Training Hyperparameters	Symbol	Numerical Value
Actor learning rate	$\eta_{\mathrm{Actor}}$	$1\times 10^{-5}$
Critic learning rate	$\eta_{\mathrm{Critic}}$	$1\times 10^{-3}$
Discount factor	γ	0.8
PER priority index	$\alpha_{\mathrm{PER}}$	0.6
PER importance sampling index	$\beta_{\mathrm{PER}}$	Starts at 0.4 and gradually increases to 1.0
Exploring noise	—	Zero-mean Gaussian noise
Maximum number of training episodes	$N_{\mathrm{cpisode}}$	200

**Table 3:** Valve experiment data.

Valve Core Stroke/mm	Average Measured Displacement/mm	Standard Deviation/mm	Maximum Value/mm	Minimum Value/mm	Error Range/mm
2	1.986	0.005	1.99	1.98	−0.02–0
4	3.982	0.008	3.99	3.97	−0.03–0
6	6.010	0.022	6.04	5.98	−0.02–0.04
8	8.008	0.019	8.03	7.98	−0.02–0.03
10	9.990	0.016	10.01	9.97	−0.03–0.01
12	11.996	0.027	12.03	11.96	−0.04–0.03
14	13.994	0.022	14.03	13.97	−0.03–0.03
16	16.010	0.022	16.04	15.98	−0.02–0.04
18	18.000	0.032	18.04	17.96	−0.04–0.04
20	20.004	0.027	20.04	19.97	−0.03–0.04

## Data Availability

The original contributions presented in this study are included in the article. Further inquiries can be directed to the corresponding author.
